# Inflammatory bowel disease addressed by Caco-2 and monocyte-derived macrophages: an opportunity for an in vitro drug screening assay

**DOI:** 10.1007/s44164-022-00035-8

**Published:** 2022-11-03

**Authors:** Sabrina Schnur, Vanessa Wahl, Julia K. Metz, Jessica Gillmann, Fabian Hans, Katharina Rotermund, Ralf-Kilian Zäh, Dietmar A. Brück, Marc Schneider, Marius Hittinger

**Affiliations:** 1 Department of Pharmacy, Biopharmaceutics and Pharmaceutical Technology, Saarland University, Saarbrücken, Germany; 2 PharmBioTec Research and Development GmbH, Saarbrücken, Germany; 3 Department of Automation, Microcontroller, Signals; School of Engineering, University of Applied Sciences, htw saar, Saarbrücken, Germany; 4 3RProducts Marius Hittinger, Blieskastel, Germany

**Keywords:** Chronic inflammation of GI-tract, Cell-based co-culture, IVIVC, Drug testing, Efficacy outcome pathways

## Abstract

Inflammatory bowel disease (IBD) is a widespread disease, affecting a growing demographic. The treatment of chronic inflammation located in the GI-tract is dependent on the severity; therefore, the IBD treatment pyramid is commonly applied. Animal experimentation plays a key role for novel IBD drug development; nevertheless, it is ethically questionable and limited in its throughput. Reliable and valid in vitro assays offer the opportunity to overcome these limitations.

We combined Caco-2 with monocyte-derived macrophages and exposed them to known drugs, targeting an in vitro-in vivo correlation (IVIVC) with a focus on the severity level and its related drug candidate. This co-culture assay addresses namely the intestinal barrier and the immune response in IBD. The drug efficacy was analyzed by an LPS-inflammation of the co-culture and drug exposure according to the IBD treatment pyramid. Efficacy was defined as the range between LPS control (0%) and untreated co-culture (100%) independent of the investigated read-out (TEER, P_*app*_, cytokine release: IL-6, IL-8, IL-10, TNF-α).

The release of IL-6, IL-8, and TNF-α was identified as an appropriate readout for a fast drug screening (“yes–no response”). TEER showed a remarkable IVIVC correlation to the human treatment pyramid (5-ASA, Prednisolone, 6-mercaptopurine, and infliximab) with an R^2^ of 0.68. Similar to the description of an adverse outcome pathway (AOP) framework, we advocate establishing an “Efficacy Outcome Pathways (EOPs)” framework for drug efficacy assays. The in vitro assay offers an easy and scalable method for IBD drug screening with a focus on human data, which requires further validation.

**Supplementary Information:**

The online version contains supplementary material available at 10.1007/s44164-022-00035-8.

## Introduction

Inflammatory bowel disease (IBD) describes a group of chronic inflammations of the gastrointestinal (GI)-tract including the two main types Crohn’s disease (CD) and ulcerative colitis (UC). With a maximal annual incidence of 20.2 per 100,000 people for CD (North America) and 24.3 per 100,000 people for UC (Europa), the disease diminishes the health and quality of life of many patients [1]. IBD is characterized by remission and acute inflammation phases, whereby, the CD is commonly affecting the ileum (small intestine) and the colon in patches through multiple layers of the tissue [2], while UC occurs as a continuous inflammation of the innermost layers (mucosa and submucosa) in the colon spreading to the rectum [3]. As the incidence for CD and UC increases, the industrial life style seems to be a prominent factor causing the occurrence of IBD [1]. However, the causes of chronic inflammations of the GI have not yet been fully identified, but multiple factors seem to have an impact [4]. Gaps in knowledge of IBD itself, the absence of a fully curative therapy and the lack of novel drug substances for an effective treatment have led to numerous animal experiments in basic research, and, above all, they are needed to fulfill safety and efficacy testing during drug development. In order to replace animal testing, an understanding of molecular mechanisms (especially the intestinal barrier) is crucial for developing an effective treatment for IBD patients. Therefore, the development and a consistent definition of an IBD in vitro assay according to the “Guidance Document For Describing Non-Guideline In Vitro Test Methods” [5] is the aim of this study. To fully investigate assay development, the main biological mechanism involved in inflammation and treatment states are important.

The intestinal barrier consists of different components, which build a strong semipermeable barrier that allows absorption of nutrients and small solutes on the one hand, but on the other hand, prevents the entering of pathogens. Primarily, the mucus layer produced and secreted by the epithelium covers the small intestine discontinuously and the colon continuously, forming the first barrier component. Secondly, the epithelium built by a cell layer that is strongly connected by tight junction (TJ) proteins is responsible for absorptive processes and simultaneously the production of mucus. Finally, cells of the immune system like monocytes/macrophages and lymphoid cells (e.g., T- and B-cells) are located in the submucosa in a “tolerant state,” ready to be activated when pathogens have crossed the mucosal and the epithelial barrier [6]. In healthy conditions, the intestinal barrier tolerates the surrounding microbiome consisting of bacteria, fungi, and viruses while the mucosa is mostly sterile and not contaminated with bacteria. In cases of CD and UC, a high bacterial contamination of the whole mucus layer and the mucosa can be observed [7]. Additionally, a “leakage” of the epithelial layer caused by the disturbance of the TJ network is reported [8]. This leads to the persistent activation of the adaptive immune system including the secretion of pro-inflammatory cytokines like tumor necrosis factor-alpha (TNF-α), which consequently sustains inflammatory conditions [9].

### Human treatment options for IBD

**Fig. 1:**
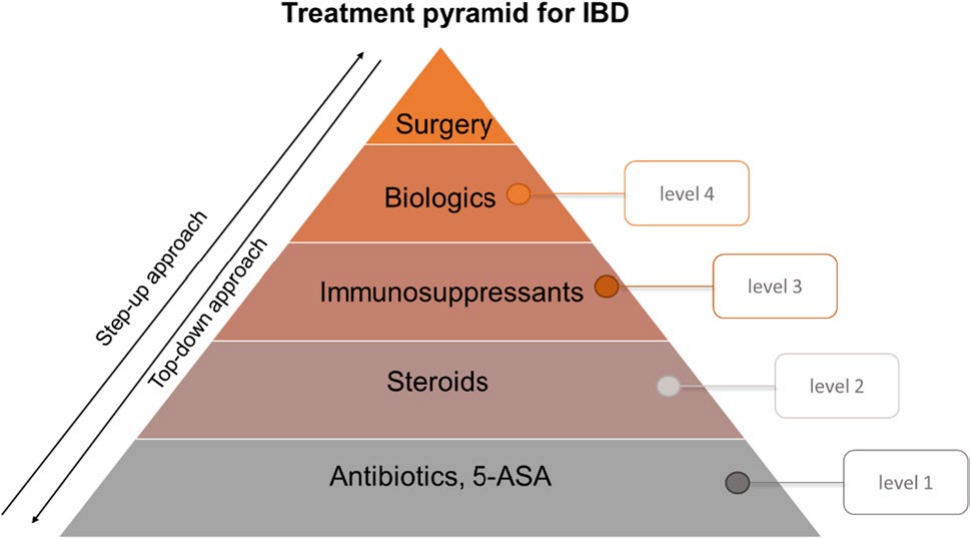
Treatment pyramid for (IBD) as step-up or top-down approach (adapted figure according to Aloi et al. [12]). Inflammatory phases with mild inflammations of the intestine are treated with antibiotics or 5-ASA derivates (level 1). Increasing severity corresponds to the usage of steroids (level 2). In severe cases, immunosuppressants (level 3) are chosen. Biologics (level 4) are used prior to surgery being considered

Presently, the cure for IBD is unknown; consequently, the therapeutic approach consists of treating the acute inflammatory phases, avoiding accompanying complications, and prolonging the remission phases. Fundamentally, the treatment is targeting the activated adaptive immune system during the acute inflammation phase of IBD. The kind of treatment applied is strongly connected to the severity of the disease [10, 11]. According to the severity level, a gradual increase in the strength of the drug treatment is widely used as depicted in the treatment pyramid (Fig. 1) [12].

Mild disease courses (level 1) are typically treated with antibiotics or 5-aminosalicylic acid (5-ASA) derivates like mesalazine [13], which intervene with the NF-κB pathway, resulting in reduced release of pro-inflammatory cytokines [14]. In mild to moderate cases (level 2), steroids like budesonide or prednisolone, that act in a similar manner on the release of pro-inflammatory cytokines by strongly inhibiting the NF-κB pathway, are applied [10, 11, 15]. For the treatment of moderate to severe disease progression (level 3), immunosuppressants like azathioprine and 6-mercaptopurine are utilized. These diminish the immune response of the body to certain antigens, especially to prolong the remission phases in steroid-dependent states. In severe cases (level 4), in which the aforementioned drugs were insufficient, biologics such as the TNF-α antibody Infliximab are deployed [10, 11]. If the efficiency of medication is limited, surgery to remove the affected regions remains as a final option [16, 17].

Based on the treatment-pyramid known drugs are able to test the prediction for the human in vivo situation of disease-related in vitro models. Due to the high rate of strong adverse effects caused by the currently administrated drugs [18], the need for new therapeutic strategies is evident. Therefore, novel drugs like JAK-inhibitors and additional antibodies have been intensively investigated [19], which are typically tested in IBD-related animal models as explained below.

### IBD-related animal models

Animal IBD models are used for the investigation of the safety and efficacy of new potential drugs in preclinical studies. Advantages of such models can be especially for chemically induced models, for instance, the simplicity in terms of inducing the inflammation, the rapidity (disease occurrence after several days after chemical administration), and controllability, as the concentration and frequency of chemical substance administration correlates with the severity of the inflammation [20–22]. In general, animal models allow for the investigation of complex physiological and biochemical interactions and modifications of the genome affecting the immune system [23, 24].

However, there are various challenges when using IBD-related animal models. One critical aspect of using chemically induced IBD models is that immune cells such as T- and B-cells are not required to induce the inflammation as occurs in humans. IBD induced by uncontrolled immune responses can be investigated with genetically engineered animals like IL-10 knockout mice [25]. Although, it is also reported that the gene depletion in different strains leads to variations in inflammation severity [26]. Crucially, the gastrointestinal physiology differs between different species [27]. However, not only is the genetic background of the used strain important to study the inflammation, many other factors, for instance, the age or gender of the animals and environmental factors influence the severity and the development time of the disease after the induction, resulting in a challenge to represent the clinical course of the disease [22]. Additionally, the induced symptoms like rectal bleeding or diarrhea cause pain, suffering, and distress for the animals which has an unknown effect on the results of the experiments [24].

Nowadays, the implementation of animal experiments is strongly connected to the ethical 3R (replacement, reduction, and refinement) principle enshrined in the European directive 2010/63. This is widely supported, for instance, by the UK-based scientific organisation NC3Rs, with the aim to replace, or at least reduce, animal experiments and to improve animal welfare [28, 29]. Consequently, reliable and validated in vitro assays as an alternative to animal experimentation are in high demand. Moreover, in vitro assays provide the potential to increase throughput for more experimental data in a reduced amount of time with the additional benefit of cost savings [24].

### Development of in vitro assay for drug screening

The purpose of the in vitro assay described here is the screening of drugs connected with human-relevant output. Based on the previous studies from co-cultures including macrophages and intestinal cells [30–36], we aimed for a co-culture which is able to address both the intestinal barrier and the immune response in IBD. As it is reported in the literature, several challenges after combining the epithelial and immune cells such as a decrease of barrier integrity when mimicking the healthy state without any stimulation [37–39] or the recovering of the inflamed barrier without any treatment or pre-stimulation of macrophages [31] occurred. In this study, we successfully developed a Caco-2/human monocyte-derived macrophages (MDM) co-culture that is able to mimic a stable healthy state and showed a significant and lasting decrease in barrier integrity measured by transepithelial electrical resistance (TEER) after stimulation with lipopolysaccharides (LPS). Therefore, this co-culture enables the evaluation of drug efficacy in vitro and provides significant benefits over previously described models. We investigated the effect of pharmaceuticals with known human efficacy according to the treatment pyramid (Fig. 1) to determine the in vitro-in vivo correlation (IVIVC) for different readouts of the assay. Finally, the in vitro assay was evaluated based on the OECD guideline “Guidance Document For Describing Non-Guideline In Vitro Test Methods” [5] to further assess the relevance of the in vitro assay and to discuss the validation that is needed to at least reduce animal experimentation in the future.

## Material and methods

The development and characterization of the co-culture were both performed by a stepwise approach. Firstly, the effects of TNF- on Caco-2 barrier properties were determined by TEER as a basis for further experimentation. Secondly, the immune response of lipopolysaccharides (LPS)-stimulated MDM was measured by analyzing the release of the cytokines tumor necrosis factor-alpha (TNF)- α interleukin (IL)-6, IL-8, and IL-10 with an enzyme-linked immunosorbent assay (ELISA). Supernatant from LPS stimulated and not stimulated MDM was then collected and added to the basolateral compartment of Caco-2 cells cultivated in a transwell system. Afterwards, the final co-culture model combining Caco-2 cells in the apical compartment and MDM in the basolateral compartment of a transwell plate was set up. The co-culture was stimulated with LPS and four different drugs, one for each severity level (according to the treatment pyramid) commonly applied to IBD patients. The co-culture system was evaluated using the parameters TEER, *P*_*app*_, and the release of TNF- α, IL-6, IL-8, and IL-10. Cytotoxicity of the drug substances was tested for Caco-2 and MDM via MTT assays.

### Cell culture

#### Cultivation of Caco-2 cells

The human colon epithelial cell line *Caco-2 HTB 37* was obtained from ATCC (American Type Culture Collection, RRID:CVCL_0025). The cells were cultivated in MEM (Minimal Essential Media, Gibco, Thermo Fisher Scientific, USA) supplemented with 20% FBS (Fetal Bovine Serum Supreme, South America origin, PAN-Biotech, Germany), 1% 100 × MEM-NEAA (MEM Non-Essential Amino Acids Solution, Gibco, Thermo Fisher Scientific, USA), 1% 100 mM Na-Pyruvate (Sodium Pyruvate, Gibco, Thermo Fisher Scientific, USA), and 1% Pen/Strep (Penicillin (10.000 units/mL)/Streptomycin (10.000 µg/mL), Gibco, Thermo Fisher Scientific, USA). The cells were maintained at 37 °C in a constant humid environment with 5% CO_2_ and were used up to passage number 50.

#### Isolation of MDM from buffy coat

For the isolation of primary MDM human blood, obtained from the Blutspendenzentrale Saar-Pfalz GmbH (Germany), was used. The blood was first diluted 1:1 with 1 × DPBS (Dulbecco’s phosphate-buffered saline, Gibco, Thermo Fisher Scientific, USA). Subsequently, the diluted blood was slowly layered above Ficoll-Paque (GE Healthcare, UK) and then centrifuged (20 min, 750 g without deceleration at room temperature (RT)) in order to break down the blood into its components. The buffy coat phase was then collected and washed twice with 1 × DPBS followed by centrifugation (7 min, 750 g at RT). The cell pellet was reconstituted in 20 mL RPMI-1640 (Gibco, Thermo Fisher Scientific, USA) supplemented with 10% hS (human Serum, heat inactivated (from male AB clotted whole blood), USA origin, Sigma-Aldrich, Merck Millipore, USA), 1% 100 × MEM-NEAA, 1% 100 mM Na-Pyruvate and 1% Pen/Strep (Penicillin (10.000 units/mL)/Streptomycin (10.000 µg/mL)). Finally, 50 ng/mL GM-CSF (Granulocyte–macrophage–colony-stimulating factor, Gibco, Thermo Fisher Scientific, USA) was added to the cell suspension, and the cells were incubated at 37 °C in a constant humid environment with 5% CO_2_. The medium was changed after 24 h, and the macrophages were used after 72 h for the experiments.

### Inflammation of monolayers

#### Inflammation of Caco-2 monolayer by TNF-α

1 × 10^5^ cells per well were seeded in the apical compartment of a transwell system (1.12 cm^2^ growth area, 12 mm transwell with 0.4 μm pore polyester membrane inserts, 12-well plate, Corning, USA). The apical and the basolateral compartment received 0.5 mL and 1.5 mL culture medium, respectively. Caco-2 cells were cultivated until a stable epithelial barrier (TEER > 500 Ω*cm^2^) was formed. The inflammation of the Caco-2 monolayer was induced by adding TNF-α (Invitrogen, Thermo Fisher Scientific, USA) in different concentrations (1 ng/mL, 2 ng/mL, 10 ng/mL, 20 ng/mL, 40 ng/mL and 60 ng/mL) to the apical and the basolateral compartment. During the experiment, the cells were maintained at 37 °C in a constant humid environment with 5% CO_2_. The effect of the stimulation on the barrier properties was monitored by TEER measurements every 24 h for 72 h.

#### Inflammation of macrophages by LPS

MDM were inflamed with LPS (lipopolysaccharides from *E. coli*, Sigma-Aldrich, Merck Millipore, USA). For this, 0.3 × 10^6^ cells/well were seeded in a 12-well plate (Greiner Bio-One International GmbH, Austria). Each well was filled with 1.5 mL MDM medium (RPMI-1640, 10% hS, 1% 100 × NEAA, 1% 100 mM Na-Pyruvate, 1% P/S). After 24 h of incubation, the macrophages were washed twice with MDM medium. Finally, 200 ng/mL LPS was added to the cells. Subsequently, the cells were incubated for 24 h at 37 °C in a constant humid environment with 5% CO_2_. The cytokine release for TNF-α, IL-6, IL-8, and IL-10 was measured by ELISA (see “Analytical methods” section “ELISA measurements”).

### Co-culture experiments

#### Stimulation with MDM supernatant

First, the supernatant of non-stimulated MDM and of MDM stimulated with LPS was used to investigate the proof of concept of the co-culture. For this, 1 × 10^5^ Caco-2 cells/well were seeded in the apical compartment of a transwell system (1.12 cm^2^ growth area, 12 mm transwell with 0.4 μm pore polyester membrane inserts, 12-well plate, Corning, USA). The apical and the basolateral compartment received 0.5 mL and 1.5 mL culture medium, respectively. The cells were cultivated until a stable epithelial barrier (TEER > 500 Ω*cm^2^) was formed. The supernatant from the macrophages was collected, and 1.5 mL/well was added to the basolateral compartment of the transwell plate with Caco-2 cells in the apical compartment. MDM medium served as control group. Cells were incubated at 37 °C in a constant humid environment with 5% CO_2_. The effect of the MDM supernatants on the barrier properties was observed with TEER-measurements every 24 h for 48 h.

#### Set-up of the MDM/Caco-2 co-culture

0.3 × 10^5^ cells/well were seeded in the apical compartment of a transwell system (0.33 cm^2^ growth area, 6.5 mm transwell with 0.4 μm pore polyester membrane inserts, 24-well plate, Corning, USA). The apical and the basolateral compartment received 0.2 mL and 0.8 mL culture medium, respectively. The cells were cultivated until a stable epithelial barrier (TEER > 500 Ω*cm^2^) was formed. The MDM were removed separately from the flask using 0.05% Trypsin–EDTA (Gibco, Thermo Fisher Scientific, USA). 0.15 × 10^6^ MDM/well were then seeded in the basolateral compartment of the transwell plate (Corning, USA). Each well was filled with 0.8 mL MDM medium (RPMI-1640, 10% FBS, 1% 100 × NEAA, 1% 100 mM Na-Pyruvate). After 24 h of incubation, the macrophages were washed twice with medium. Finally, the transwell inserts containing the Caco-2 cells were set above the macrophages located in the basolateral compartment. The co-culture was maintained at 37 °C in a constant humid environment with 5% CO_2_. The barrier properties of the co-culture were monitored by performing TEER-measurements after 4 h, 24 h, and 48 h.

#### Inflammation of co-culture

To induce an inflammation of the co-culture, LPS was added basolaterally to each well (final concentration per well: 200 ng/mL) directly after the co-culture was initiated. TEER-measurements were carried out after 4 h, 24 h, and 48 h.

#### Proof of concept study for efficacy verification of commonly applied drugs for IBD patients

For the treatment of the inflamed cell state, the four different drugs: the 5-amino salicylic acid derivate mesalazine (Cayman Chemical Company, USA), the corticosteroid prednisolone (TCI Deutschland GmbH, Germany), the immunosuppressant 6-mercaptopurine (TCI Deutschland GmbH, Germany), and the TNF-α antibody infliximab (Merck Millipore, USA) were selected, which are commonly used for the treatment of IBD. After applying the respective drug to the in vitro model, the treatment efficacy of each drug was investigated. A drug concentration of 200 µg/mL was chosen empirically as a high dose with non-cytotoxic effects. The dose is expected to be much higher than the in vivo exposure and is sufficient for in vitro proof of concept investigations. Dose findings in vitro with a transfer to in vivo data are a future challenge.

#### Treatment of MDM

After isolating MDM from buffy coat as described in Sect. Cell culture, the cells were seeded on a 12-well plate (Greiner Bio-One International GmbH, Austria). For this, the MDM were removed from the flask using 0.05% Trypsin–EDTA (Gibco, Thermo Fisher Scientific, USA). 0.15 × 10^6^ MDM/well were then seeded on the 12-well plate. Each well was filled with 1.5 mL MDM medium (RPMI-1640, 10% FBS, 1% 100 × NEAA, 1% 100 mM Na-Pyruvate). After 24 h of incubation, the macrophages were washed twice with the medium. Subsequently, MDM were exposed to the selected drug suspensions in a concentration of 200 µg/mL (volume: 1.5 mL). Immediately afterwards, LPS were applied to all wells (final concentration 200 ng/mL) except for the medium control. LPS-stimulated MDM that were not treated with drugs were used as positive control. The cells were maintained at 37 °C in a constant humid environment with 5% CO_2_. After 24 h, the supernatants of the cells were collected and the cytokine release was analysed by ELISA (see “Analytical methods” section “ELISA measurements”).

#### Treatment of the MDM/Caco-2 co-culture

After setting up the co-culture, the selected drugs were applied in a final concentration of 200 µg/mL to each basolateral compartment to the macrophages. MDM medium (RPMI-1640, 10% FBS, 1% 100 × NEAA, 1% 100 mM Na-Pyruvate, 1% P/S) was used as a negative control (no inflammation). Immediately afterwards, LPS in a final concentration of 200 ng/mL were added to all wells in the basolateral compartment except for the negative control (medium control). Macrophages that were stimulated with LPS but not treated served as positive control (inflammation). The co-culture was maintained at 37 °C in a constant humid environment with 5% CO_2_. Treatment efficacy was investigated by doing TEER measurements after 4 h, 24 h, and 48 h followed by a transport study with sodium fluorescein. The cytokine release of the MDM was investigated by performing ELISA (see “Analytical methods” section “ELISA measurements”).

### Analytical methods

#### MTT assay

The cell viability after exposure to the selected drugs was investigated by MTT assays as described by Scherließ et al. [40]. In brief, the cells (4 × 10^4^ Caco-2 cells or 2.72 × 10^4^ MDM) were seeded in a 96-well plate (Greiner Bio-One International GmbH, Austria) using a volume of 200 µL/well. The cells were incubated for 24 h at 37 °C in a constant humid environment. One day before the experiment, the dilutions of the drugs were prepared. For Mesalazine (Cayman Chemical Company, USA), prednisolone (TCI Deutschland GmbH, Germany) and 6-Mercaptopurin (TCI Deutschland GmbH, Germany) concentrations from 0.005 mg/mL up to 5 mg/mL and for infliximab (Merck Millipore, USA) from 0.005 mg/mL up to 2 mg/mL were chosen. The dilutions were prepared in 1 × HBSS (Hanks’ Balanced Salt Solution, Gibco, Thermo Fisher Scientific, USA). To remove medium residues, the cells were washed twice with 200 µL HBSS, respectively. Afterwards, 200 µL of the respective drug concentration was applied to the cells followed by an incubation on a shaker at 37 °C and 35 rpm for 4 h. Onefold-concentrated HBSS (100% viability) and Triton-X-100 (AppliChem GmbH, Germany) (0% viability) were used as controls. The supernatant was then disposed, and the cells were washed once with 1 × HBSS. Subsequently, the MTT reagent ((3-(4,5-dimethylthiazol-2-yl)-2,5-diphenyl tetrazolium bromide, Agros Organics, Thermo Fisher Scientific, USA) was added in a concentration of 0.5 mg/mL per well, and the cells were incubated again on a shaker for 4 h at 37 °C and 35 rpm, protected from light. The MTT reagent was then removed and 100 µL DMSO was added to the wells, respectively, incubated for 15 min on a shaker at 37 °C and 35 rpm, again protected from light. Finally, the absorbance was measured at 550 nm with the Synergy2 plate reader (BioTek Instruments GmbH, USA). The viability of the cells was determined by the following formula:

1 $$Viability\;\left[\%\right]=\frac{{absorbance}_{test\;formulation}-{absorbance}_{TritonX-100}}{{absorbance}_{HBSS}-{absorbance}_{TritonX-100}}\ast100$$

Formula 1 describes the calculation of cell viability in %.

#### TEER measurements

The TEER-values of the Caco-2 monolayer were measured with the epithelial voltohmmeter EVOM2 (World Precision Instruments, USA) together with the STX2 Chopstick Electrode Set (World Precision Instruments, USA). First, the electrodes were cleaned with 70% isopropanol, dried, and then placed in the apical or basolateral compartment, respectively.

#### Transport studies

The transport studies were performed using sodium fluorescein as a marker substance. For this, medium of Caco-2 cells was replaced by 1 × HBSS (Gibco, Thermo Fisher Scientific, USA) and the cells were incubated for 30 min at 37 °C. Subsequently, the initial TEER values were measured before 10 µg/mL sodium fluorescein (final concentration apical); solution was applied to the cells in each well in the apical compartment. The experiment was performed at 37 °C under constant shaking (30 rpm) and light protection. Samples were taken from the basolateral compartment after 0 min, 20 min, 40 min, 60 min, 90 min, and 120 min and from the apical compartment after 0 min and 120 min. To ensure that the epithelial barrier was not damaged during the experiment, the TEER values of the cells were measured at the end of the experiment. The absorbance of the samples was analysed at 528 nm with the Synergy2 plate reader (BioTek Instruments GmbH, USA). Finally, the apparent permeability coefficient (*P*_*app*_) values were determined by the following formula:

2 $$Papp=\frac{\Delta q}{\Delta t}*\frac{1}{{c }_{0 }(apical)}*\frac{1}{{A}_{Transwell}}$$

Formula 2 describes the calculation of the apparent permeability coefficient *P*_*app*_ (cm/s)*.* Δ*q*/Δ*t* is the mass transport over time [µg/s], *c*_0_ is the initial concentration of sodium fluorescein [µg/mL], and *A*_Transwell_ is the area of the transwell membrane (1.12 cm^2^).

#### ELISA measurements

For the investigation of the cytokine release of the macrophages in stimulated or non-stimulated conditions, the Human Uncoated ELISA Kits for TNF-α, IL-6, IL-8, and IL-10 from Thermo Fisher Scientific, USA were used. The assay was performed according to the manufacturer’s protocol. Briefly, 96-well plates (Corning, USA) were coated with a 100 µL/well capture antibody. The plates were incubated at 4 °C overnight. Each well was then washed three times with 200 µL/well wash buffer, composed of 1 × DPBS containing 0.05% polysorbate-20 (Merck Millipore, USA) before the wells were blocked with 100 µL/well 1 × ELISA spot diluent for 1 h at room temperature. After the incubation, the wells were washed once with 200 µL/well wash buffer. The samples (100 µL/well) were first centrifuged for 5 min at 0.8 g and were then applied to the wells in the following dilutions: 1:1 and 1:100 for TNF-α samples, 1:100 and 1:1000 for IL-8 samples, and undiluted and 1:100 for IL-6 samples. The standards thus provided were used to obtain a calibration curve for each tested cytokine. The plates were again incubated at 4 °C overnight. The next day, each well was washed five times and 100 µL/well detection antibody was added followed by an incubation for 1 h at room temperature. Subsequently, the wells were washed again five times and then 100 µL/well Avidin-horseradish peroxidase (HRP) was applied, followed by an incubation for 30 min at room temperature. After washing six times, 100 µL/well 3, 3′, 5, 5'-tetramethylbenzidine (TMB)-substrate was added and the wells were incubated for 15 min at room temperature. Finally, 100 µL/well stop solution (1 M ortho-phosphoric acid, VWR International, USA) was used to stop the enzymatic reaction. The samples were measured at 450 nm and 570 nm with the Synergy2 plate reader.

#### ZO-1 staining of Caco-2 monolayer

To visualize the barrier-forming protein zonula occludens (ZO)-1, the Caco-2 cells were fixed and stained as described in the following. First, Caco-2 cells were washed with 0.5 mL 1 × DBPS (Gibco, Thermo Fisher Scientific, USA) apical and 1.5 mL 1 × DPBS basolateral. The cells were fixed with ice-cold methanol (VWR International, USA). 0.5 mL was added apical and 1.5 mL basolateral for 5 min at 4 °C. Washing with 1 × DPBS was repeated three times. Afterwards, a blocking step with 0.5 mL 1% BSA (Thermo Fisher Scientific, USA) in the apical compartment was done for 30 min at 4 °C. Subsequently, 1 µL ZO-1 primary antibody in a final concentration of 2 µg/mL was added to each well filled with 0.5 mL 1% BSA, respectively. The cells were incubated overnight at 4 °C, protected from light. After that, the cells were washed three times with 1 × DPBS with an incubation time of 5 min at each step at RT. Then, 1 µL Alexa Fluor 488 goat anti rabbit IgG (Thermo Fisher Scientific, USA) in a final concentration of 2 µg/mL was added to 0.5 mL 1% BSA, followed by an incubation over night at 4 °C, protected from light. Afterwards, the cells were washed twice with 0.5 mL DPBS with an incubation time of 5 min at RT for each washing step. The nuclei of the Caco-2 cells were stained with 1 µL 4′, 6-diamidino-2-phenylindole (DAPI) (Thermo Fisher Scientific, USA) in a final concentration of 2 µg/mL. Each well was filled with 0.5 mL 1 × DPBS followed by an incubation for 15 min at RT. Finally, the cells were mounted with mounting medium (Sigma-Aldrich, Merck, USA) and were imaged via confocal laser scanning microscopy (LSM710, Zeiss, Oberkochen, Germany).

### Statistical analysis

The statistics were performed with the program OriginPro 2021. As different numbers (n) of independent replicates were performed, the data is presented as the mean ± standard deviation (SD). For the statistical analysis, one-way analysis of variance (ANOVA) and the post hoc Bonferroni test were used. A two-sampled t-test was applied whenever two groups were compared.

## Results

### Inflammation of monolayers

#### Inflammation of Caco-2 monolayer by TNF-α

Due to the expression of tight junction proteins (see Supplementary information, ZO-1 staining Caco-2 monolayer Fig. S1), the Caco-2 cells formed a stable epithelial barrier (TEER > 500 Ω*cm^2^). After the application of TNF-α in different concentrations, a dose-dependent decrease of the TEER values (see Supplementary information, Fig. S2) was measured, indicating a loss of barrier integrity. The maximal effect could be observed after stimulation with 10 ng/mL TNF-α. Higher concentrations of TNF-α up to 60 ng/mL did not result in a higher decrease of TEER values.

#### Inflammation of macrophages by LPS

The stimulation of MDM with 200 ng/mL LPS led to an increased release of the cytokines TNF-α, IL-6, and IL-8 compared to the medium control (see Supplementary information, Fig. S3), which demonstrates a successfully induced inflammation of the macrophages. As the MDM were freshly isolated from the human buffy coat for each experiment, the cytokine release of the MDM showed an increased variance between different donors. However, a clear difference in the measured cytokine profile of the non-inflamed and inflamed condition was nevertheless observable.

### Set-up and LPS stimulation of MDM/Caco-2 co-culture

**Fig. 2:**
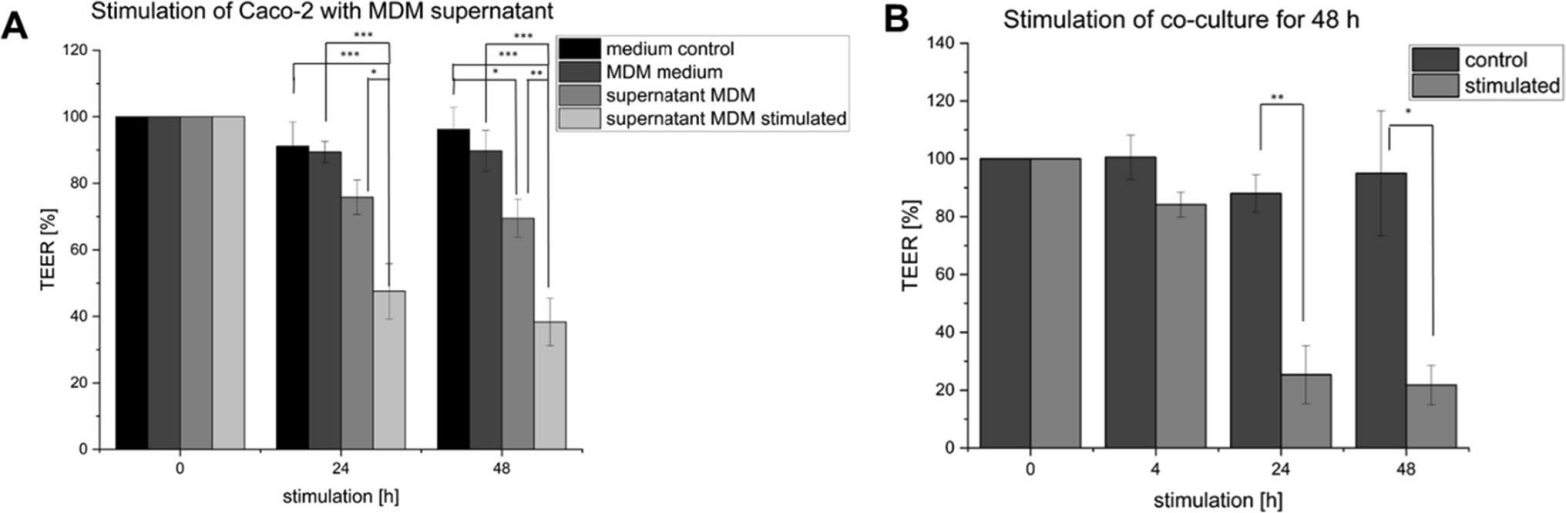
Effect on barrier properties investigated by TEER measurements after incubation with different stimuli. The Caco-2 cells stimulated with the supernatant of LPS-stimulated MDM resulted in a significant decrease of TEER to 38% of the initial value while the stimulation with non-stimulated MDM supernatant showed only a slight decrease. Results are represented as mean ± SD for in summary *n* = 9 wells for each group performed in 3 biological replicates (**A**). In the co-culture system, stable TEER values could be obtained for the combination of Caco-2 and MDM without any stimulation (= medium control), which indicates an intact epithelial barrier. The LPS-stimulation of the MDM in the basolateral compartment induced a significant decrease in TEER. Results are represented as mean ± SD and for in summary *n* = 18 wells for each group performed in three biological replicates (**B**). *p < 0.05, **p < 0.01, and ***p < 0.001 indicate significant difference

The set-up of the MDM/Caco-2 co-culture was first assessed by only applying the supernatant from non-stimulated or with LPS-stimulated macrophages in the basolateral compartment of the Caco-2 cells. The TEER measurement over 48 h showed a slight decrease in TEER after the application of non-stimulated MDM supernatant compared to the medium control. The supernatant from stimulated macrophages led to a significant decrease to 38% of the initial TEER value (Fig. 2A). The combination of MDM and Caco-2 in a co-culture system was characterized by a stable TEER for the control group without any stimulation and a significant drop in TEER to 21% after 48 h of LPS stimulation (Fig. 2B).

### Treatment of the co-culture

The MDM/Caco-2 co-culture was treated with four different drugs, which correlate with the treatment-pyramid for IBD (Fig. 1, section “Human treatment options for IBD”). Depending on the severity level, different types of drugs are commonly applied to the patients to cure acute inflammations and to maintain remission phases of the disease. As a first step in treating the MDM/Caco-2 co-culture, the viability of the two cell types after incubation with the active ingredients was investigated by MTT assays to exclude cytotoxic effects. Concentrations up to 2 mg/mL or 10 mg/mL, depending on the drug, can be applied to the cells without decreasing the cell viability (see Supplementary information, Figs. S4 and S5).

#### Effect on barrier integrity (Readout: TEER, P_app_)

**Fig. 3:**
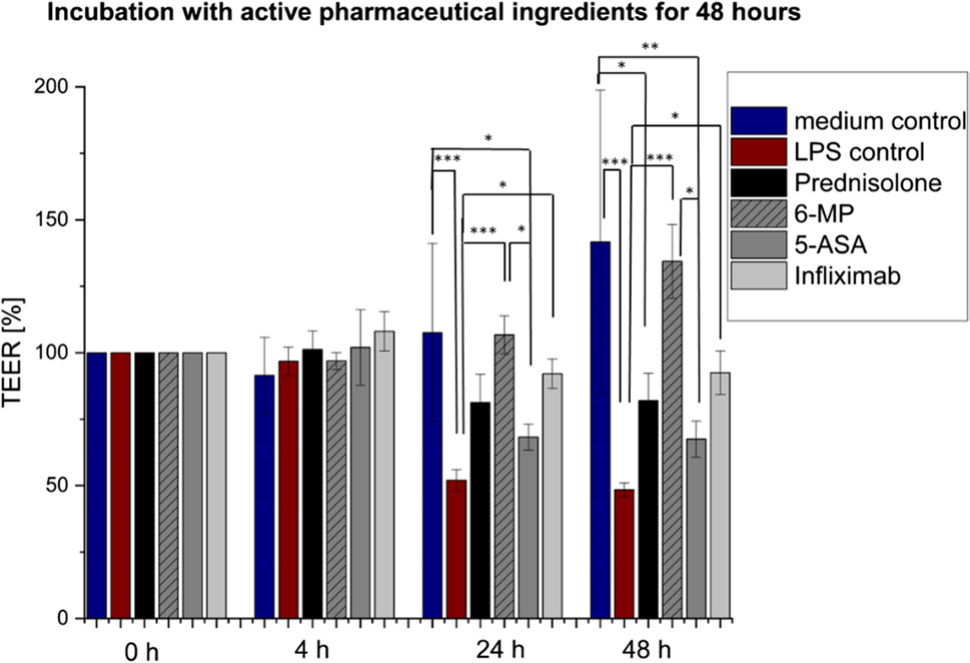
Investigation of drug efficacy for 5-ASA, prednisolone, 6-MP, and infliximab on the barrier integrity of the MDM/Caco-2 co-culture observed by TEER measurements for 48 h. The strongest stabilization effect on the barrier integrity could be reached after the treatment with the immunosuppressant 6-MP (no difference to medium control), whereas TNF-α antibody infliximab maintained 92% of TEER. The Caco-2 cells affected with 5-ASA and prednisolone showed still higher TEER values in comparison to the LPS control. Results are represented as mean ± SD for in summary *n* = 10 wells for each group performed in five biological replicates with *n* = 2 wells. *p < 0.05, **p < 0.01, and ***p < 0.001 indicate significant difference

The drugs’ effect on the integrity of the barrier was investigated with TEER measurements for 48 h followed by transport studies with sodium fluorescein. The immunosuppressant 6-MP has no significant effect on TEER compared to the medium control, unlike on the LPS control after 24 h (Fig. 3). The treatment with the TNF-α antibody infliximab resulted in a stable TEER of 92% after 48 h compared to the initial value and showed a significant difference to the LPS control where a decrease in TEER to 48% could be observed. The corticosteroid prednisolone reduced the LPS-induced TEER decrease to 82% of the initial value. The slightest effect could be measured for the treatment with the 5-ASA derivate mesalazine, where the LPS-induced TEER decrease was stabilized to 67% of the initial value after 48 h.

Transport studies with sodium fluorescein that were performed to investigate the permeability of the epithelial barrier yielded *P*_*app*_ values in an interval from 9.70 × 10^−8^ cm/s to 1.11 × 10^−6^ cm/s with no visible differences for the respective groups (see Supplementary information, Fig. S6), indicating no increased permeability of the epithelial barrier.

#### Effect on cytokine release of MDM

**Fig. 4:**
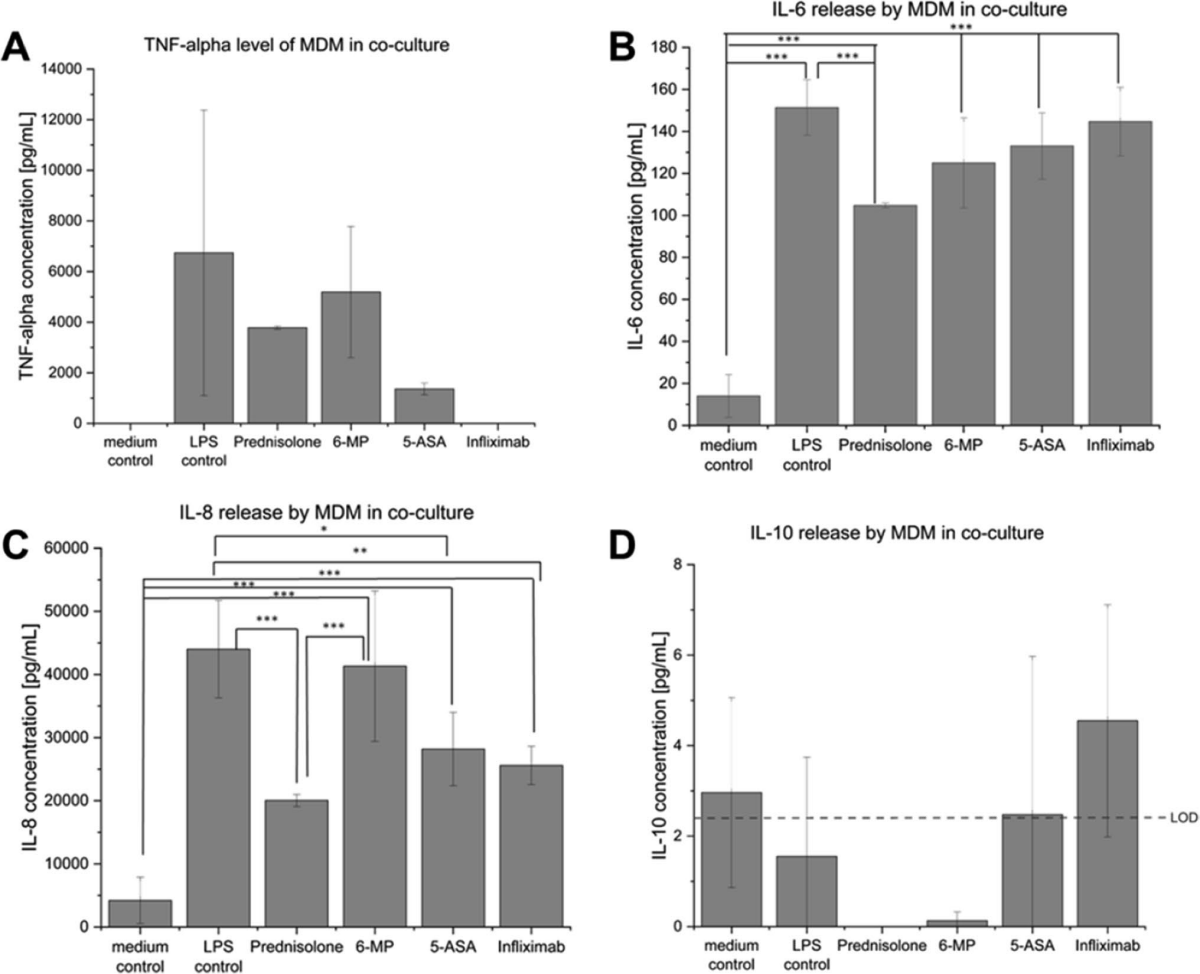
Cytokine release by MDM in the co-culture system after 48 h after the treatment of 5-ASA, prednisolone, 6-MP, and infliximab. **A** TNF-α: as a TNF-α antibody infliximab inhibited the TNF-α release by the macrophages. Prednisolone and 6-MP only had a slight effect on the release compared to the LPS control, whereas 5-ASA had a stronger effect. **B** IL-6: the release of IL-6 could be significantly reduced in comparison to the LPS control by the treatment with prednisolone, whereas 5-ASA, 6-MP, and infliximab only showed a small effect. **C** IL-8: a significant decrease of the secreted IL-8 was achieved after the treatment with 5-ASA, prednisolone and infliximab. **D** IL-10: the overall IL-10 content was low in comparison to the pro-inflammatory cytokine release. The highest release of IL-10 was observed after the treatment with infliximab. *LOD* = limit of detection. Results are represented as mean ± SD for in summary *n* = 6 wells for each group performed in three biological replicates. *p < 0.05, **p < 0.01, and ***p < 0.001 indicate significant difference

The release of the cytokines TNF-α, IL-6, IL-8, and IL-10 by the MDM in the co-culture system after 48 h of stimulation and drug treatment was analysed by ELISA. No level of TNF-α was observed after the treatment with the TNF-α antibody infliximab (Fig. 4A). The supernatant contained 3786 pg/mL and 5191 pg/mL of TNF-α after prednisolone and 6-MP treatment, which showed only a slight effect on the TNF-α release in comparison to the LPS control, which had a TNF-α content of 6741 pg/mL. The treatment with 5-ASA resulted in a reduced cytokine release to 1362 pg/mL. Figure 4B presents the results for the IL-6 release, only prednisolone reduced the IL-6 release significantly to 104 pg/mL compared to the LPS control containing 151 pg/mL IL-6. For the IL-8 release a significant decrease could be measured after the treatment with 5-ASA (28,190 pg/mL), prednisolone (20,048 pg/mL), and infliximab (25,603 pg/mL) compared to the LPS control (44,024 pg/mL) (Fig. 4C). The investigation of the anti-inflammatory cytokine IL-10 showed almost no release with the highest measurable values of 2.96 pg/mL for the medium control and of 4.55 pg/mL for the treatment with infliximab (Fig. 4D).

### Evaluation of readout parameter with focus on drug efficacy

**Fig. 5:**
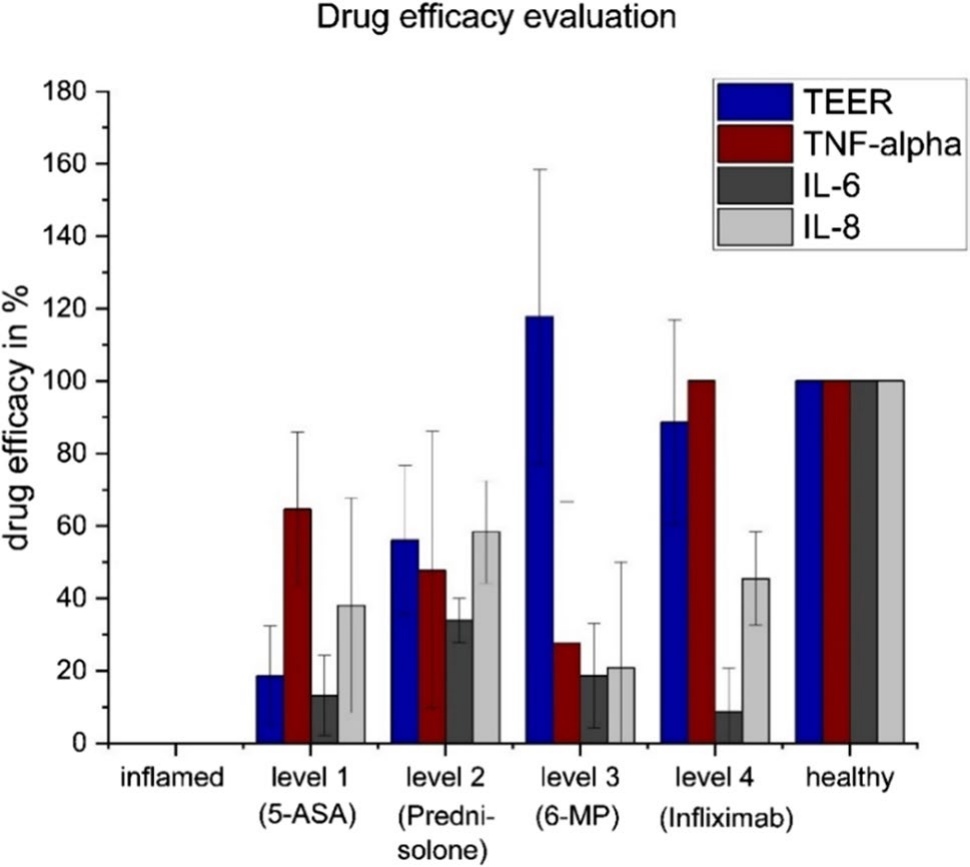
Assessment of in vitro drug efficacy for the readouts TEER, TNF-α, IL-6, and IL-8. The drug efficacy was calculated in percent relative to the medium control (healthy, 100% drug efficacy) and the LPS control (inflamed, 0% drug efficacy) against the severity level represented by the respective drugs. The readouts TEER, TNF-α, IL-6, and IL-8 were used for the evaluation of drug efficacy with the co-culture model. Results are represented as mean ± SD for in summary *n* = 10 wells for each group performed in five biological replicates for the TEER measurements and for in summary *n* = 6 wells for each group performed in three biological replicates for the cytokines

To evaluate the proof of concept for the in vitro drug efficacy for the above-mentioned drugs, based on the known efficacy in IBD patients, the measured values in the co-culture model for each readout: TEER, TNF-α, IL-6, and IL-8 were calculated in percent relative to the medium control. No inflammation is indicated by 100% drug efficacy (medium control) and a complete inflamed state by 0% drug efficacy (LPS stimulation). Concerning the read-out *P*_*app*_ no influence of stimulation or drug treatment (see Supplementary information, Fig. S6) was visible, and for IL-10 the release was very low (maximum of 4.55 pg/mL, Fig. 4D), so they were not included in the drug efficacy evaluation. The results displayed in Fig. 5 are plotted against the severity levels 1 to 4 of the disease as represented by the selected drugs. TEER, TNF-α, IL-6, and IL-8 all produced positive responses and are thus qualified as potential readouts for an IVIVC which will be investigated in the next sections.

### Correlation with severity level

#### Readout: cytokine release

The calculated minimal and maximal drug efficacy for the readouts TNF-α, IL-6, and IL-8 are summarized in Table 1. The drug efficacy depended on the type of drug and differed in the analysed cytokine releases. In terms of TNF-α, the minimal drug efficacy of at least 28% was observed for the immunosuppressant 6-MP, while the TNF-α-antibody infliximab showed an efficacy of 100%. A mean efficacy of 60 ± 44% for the tested drugs was calculated. The treatment resulted in a minimal drug efficacy for IL-6 of 9% for infliximab and a maximum of 34% for prednisolone. The calculated mean drug efficacy for IL-6 was 19 ± 50%. For the reduction of IL-8 release, prednisolone was the most effective drug with an efficacy of 68%, and 6-MP was the least effective drug with an efficacy of 21%. The mean drug efficacy for IL-8 was 41 ± 19%. Based on these observations, the cytokine release, especially for TNF-α, is a responsive readout for the drug treatment and offers the possibility to investigate “yes–no”-responses from the co-culture model to test the efficacy of drugs.

**Table 1:** Summary of the calculated minimal, maximal, and mean drug efficacy for TNF-α, IL-6, and IL-8. The analysis of the cytokine release allows a “yes–no” response from the co-culture for the drug efficacy, which depends on the applied drug and the analysed cytokine. Mean drug efficacy was calculated as mean ± SD for the different drugs out of in summary *n* = 6 wells for each group performed in three biological replicates

	TNF-α	IL-6	IL-8
Minimal drug efficacy	28% (6-MP)	9% (infliximab)	21% (6-MP)
Maximal drug efficacy	100% (infliximab)	34% (prednisolone)	58% (prednisolone)
Mean drug efficacy	60 ± 44%	19 ± 50%	41 ± 19%

#### Readout: TEER

**Fig. 6:**
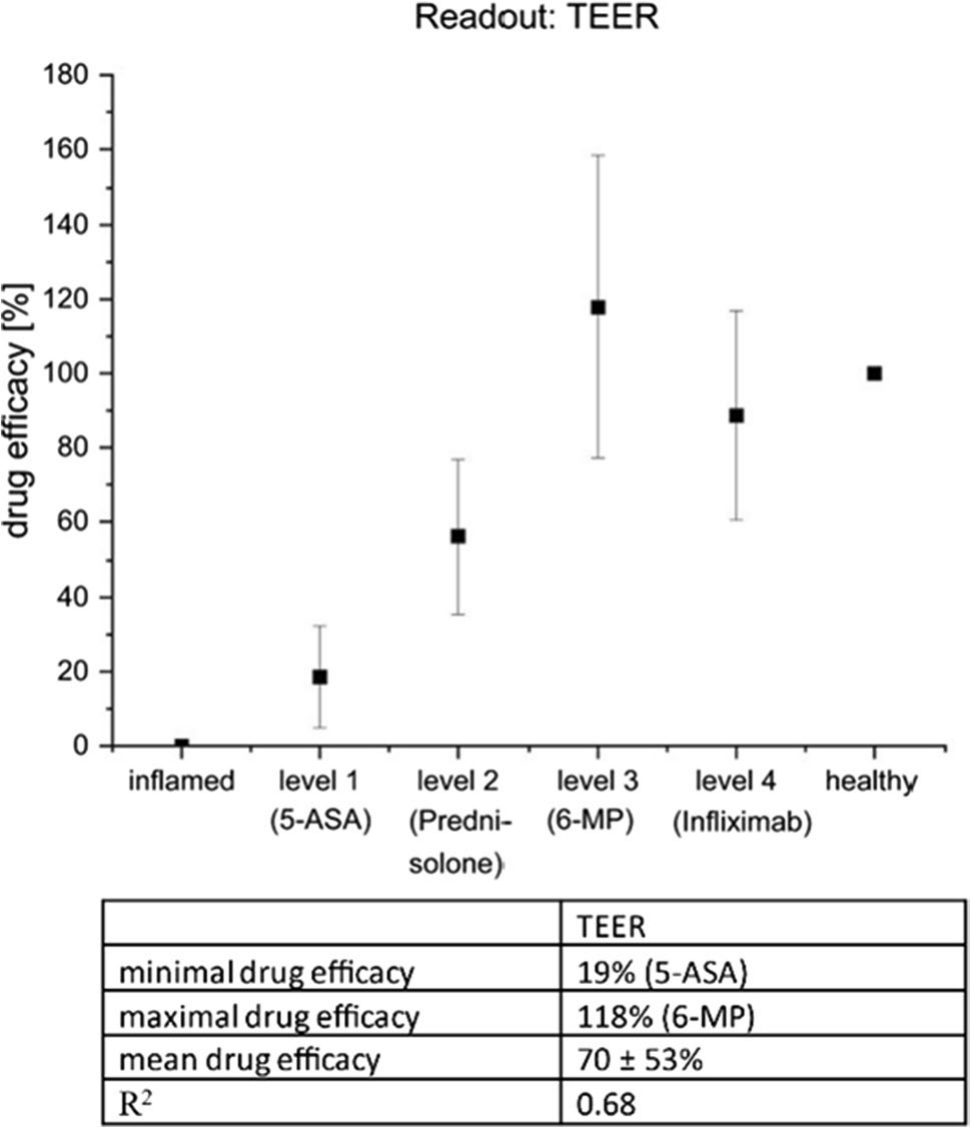
Plot of calculated responses in % for the drug efficacy investigated by TEER measurements against the severity level represented by the respective drugs. The response increases from ASA (level 1) to prednisolone (level 2) up to 6-MP (level 3). Infliximab (level 4) had a slight decrease in response in comparison to 6-MP (level 3). The correlation of TEER with the severity level of the disease resulted in a R^2^ of 0.68. Results are represented as mean ± SD for in summary *n* = 10 wells for each group performed in five biological replicates

The plot against the increasing severity level represented by the respective drug for the readout TEER is shown in Fig. 6. The treatment response increases from 19% for 5-ASA (level 1) to 56% for prednisolone (level 2) and up to a 118% response for 6-MP (level 3). The alternative biological drug infliximab (level 4) showed a slight decrease in response to 89% in comparison to 6-MP (level 3). The correlation of TEER with the severity level of the disease resulted in a R^2^ of 0.68. Based on this, a possible chance for IVIVC, additional to the yes–no response for drug efficacy, is conceivable, when TEER as a readout is investigated.

## Discussion

### In vitro assay development

The in vitro assay was developed following a stepwise approach. Firstly, the suitability of the human colorectal adenocarcinoma cell line Caco-2 was investigated in terms of its change in barrier properties after stimulation with the pro-inflammatory cytokine TNF-α in different concentrations. The stimulation with 10 ng/mL TNF-α showed the maximum TEER decrease, which is in accordance with previously reported data by Ma et al. [41]. The advantage of working with Caco-2 cells is their good availability and culture constancy, as well as their ability to form a stable tight junctions network measurable via TEER, the spontaneous differentiation into polarized enterocytes, and the good correlation to the human tissue especially in terms of permeability and drug absorption [42–44]. Additionally, as expected, the effect of stimulation with TNF-α, a key mediator in inflammatory bowel disease [45, 46], resulted in a disturbance of the tight junctions network. Therefore, the reduction of barrier properties similar to IBD has been regularly reported [8, 47, 48]. However, the Caco-2 monolayer alone is not able to mimic the complex cell interactions in vivo, especially the important role of immune cells in IBD [19].

Secondly, the macrophages play a central role in the innate immune system of the intestine by accepting the natural microbiome, being able to react quickly to environmental changes by adapting their phenotype, and fighting entering pathogens [49, 50]. Therefore, in this study, MDM were isolated from human blood and characterized in terms of cytokine release in an inflammatory state, induced by LPS stimulation. For the purpose of differentiation, GM-CSF was used which stimulates the differentiation of monocytes to macrophages designated as M1-cells [49]. Human GM-CSF macrophages provide the ability to release high levels of Th1-cytokines (e.g., IL-12 and IL-23) and other pro-inflammatory cytokines such as TNF-α and IL-6. Besides, they release very low to undetectable levels of IL-10 [49] [51], which could be verified in this study by measuring no IL-10 release of the MDM, indicating the M1-type. While in the non-stimulated state of MDM, no levels of TNF-α and IL-6 and only a very low release of IL-8 was measured in the co-culture, the LPS stimulation led to an increased release of the previously mentioned cytokines, which is typical for GM-CSF macrophages [51] [52]. Despite the donor dependency observed in the experiments, the human origin of MDM and the high release of pro-inflammatory cytokines offer a chance for an increased IVIVC.

Similar to the in vivo situation, in which the MDM are located directly under the epithelium [50], the cells were seeded in the basolateral compartment of a transwell system and combined with the Caco-2 cells grown in the apical compartment. The combination of intestinal epithelial cells and macrophages was addressed in various in vitro models that allow studying the cell interaction of epithelial and immune cells in healthy and inflamed conditions [30–32, 53]. However, challenges in combining differentiated macrophages with epithelial cells in a co-culture system, affecting the barrier integrity without any further stimulation, were also reported [37–39]. In this study, the Caco-2 cells accepted the medium of MDM measurable as stable TEER values (Section “Set-up and LPS stimulation of MDM/Caco-2 co-culture”, Fig. 2A). Even in the presence of the MDM in the basolateral compartment, the barrier properties of the Caco-2 cells remained stable over the tested time period of 48 h (Section “Set-up and LPS stimulation of MDM/Caco-2 co-culture”, Fig. 2B). The stimulation with supernatant of LPS-stimulated MDM led to significant decreases in TEER values after 24 h indicating the presence of inflammatory processes (Fig. 2A). In our co-culture set-up, the inducing of inflammation with LPS resulted in a significant TEER drop of 52% after 48 h (Fig. 2B). The four drugs, mesalazine, prednisolone, 6-mercaptopurin, and infliximab, which, in that order, correspond to the severity levels 1 to 4 as presented in the treatment pyramid (Fig. 1), were applied as proof of concept for evaluating the drug efficacy. The treatment with mesalazine recovered the epithelial barrier to 67% of the initial TEER value, prednisolone to 82%, 6-mercaptopurine fully recovered the barrier, and infliximab did so to 92% of the initial TEER value. The treatment with mesalazine, prednisolone and infliximab also significantly decreased the IL-8 release of the immune cells in comparison to the LPS control.

The efficacy testing of drugs using in vitro models mimicking IBD was previously reported. For instance, Leonard et al*.* developed a 3D-co-culture model out of human blood-derived macrophages, dendritic, and intestinal epithelial cells embedded in a collagen layer for drug screening addressing the treatment of IBD. The IL-1β induced inflammation led to a drop in TEER of 10 to 20% after 48 h. The treatment with different budesonide (nano)formulations (except of liposomal formulation) recovered the intestinal barrier and free budesonide and a PLGA-budesonide formulation decreased the IL-8 release [54]. The effect of siRNA-based nano-medicine was tested with the “leaky gut” model that consists of Caco-2 cells, THP-1 macrophages, and MUTZ-3 cells, developed by Hartwig et al*.* The negatively affected intestinal barrier in IBD was mimicked by performing the treatment experiments just before the Caco-2 cells reached confluency resulting in TEER values below 200 Ω*cm^2^. The LPS stimulation of the leaky model showed comparable higher release of the pro-inflammatory cytokines IL-8 and TNF-α [35], which was also observed in our experiments. The comparable strong TEER decrease in the inflammatory state, that was successfully obtained with our model, allows for drug efficacy screening of different drugs with variable strongness and therefore for IVIVC evaluation, which is highly necessary when developing an in vitro assay according to the 3R principle.

### Evaluation of in vitro assay readout

**Fig. 7:**
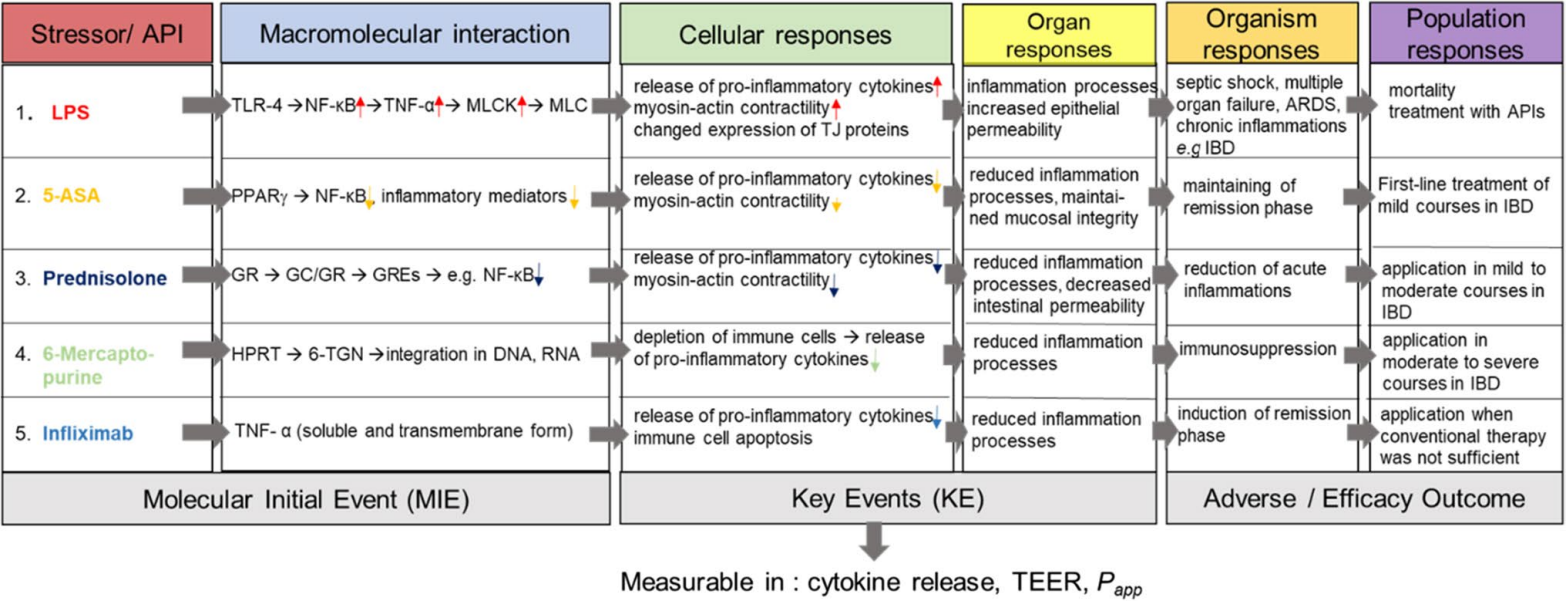
Tested compounds and their in vivo response presented as adverse outcome pathway (AOP) for LPS and efficacy outcome pathway (EOP) for the APIs. Starting with the molecular initial event (MIE), the binding to the biological target, followed by a series of essential biological events, including the measurable key events (KE) cytokine release, TEER, *P*_*app*_ and with the adverse outcome (AO)/efficacy outcome (EO) that represents the endpoint

Data obtained from assay development have to be discussed in the context of the “Guidance Document For Describing Non-Guideline In Vitro Test Methods” in order to evaluate the possibility for an IVIVC [5]. Therefore, the in vitro model was described based on the idea of the AOP framework, where tested compounds and their (toxic) in vivo response are presented. The AOP framework is described by the OECD as an analytical construct that combines a series of successive events on different compartments of the organism that are causally linked, focused on the critical steps [55, 56]. The starting point of the AOP framework is the molecular initial event (MIE), such as the initial effect of the interaction of a stressor/active compound with the biological target. The process continues with a series of essential biological activities or pathways and ends with the final adverse outcome (AO) in the organism. Key events (KE) express the measurable essential changes or reactions on a cellular or physiological level [55, 56]. In this study, the efficacy of IBD-related drugs was evaluated; consequently, the active compound here is an active pharmaceutical ingredient (API). The in vivo effect of the APIs on macromolecular interactions, cellular, organ, organism, and population responses was therefore defined as “Efficacy Outcome Pathway (EOP).” In contrast to this, the stressor LPS, which is used to induce the inflammation, is described as a typical AOP. The readouts of the in vitro assay are clearly located on the cellular response level (KE)(Fig. 7).

The first MIE, described along essential steps of the pathway through the AOP framework, is the LPS-binding to toll-like receptor 4 (TLR-4) which activates a signal transduction via two principal signalling pathways (MyD88-or TRIF-mediated) that results in NF-κB activation which further leads to induction of TNF-α-mRNA [57–59]. As a consequence, TNF-α enhances the myosin light chain kinase (MLCK) expression, which invokes a hyperphosphorylation of the myosin light chain (MLC) followed by an enhanced myosin-actin contractility [60]. Besides, the activation of the NF-κB pathway changes the expression level of tight junctions proteins such as claudin and occludin proteins [61, 62]. As a result, the TJ protein network is disturbed and the permeability of the epithelial barrier is increased [60] [62]. The organism response consists of septic shock, multiple organ failure, acute respiratory distress syndrome (ARDS), and inflammatory diseases like IBD [63–65].

One important KE is the release of not only TNF-α, but numerous pro-inflammatory cytokines such as IL-6 or IL-1 and different cytokines from human MDM as LPS response [58, 66]. In the in vitro assay, the release of the pro-inflammatory cytokines TNF-α, IL-6, and IL-8 was measured by performing ELISA measurements. A significant increase in the release of the respective cytokines could be observed after LPS stimulation of the co-culture systems, which indicates the sensitivity of the in vitro assay for this KE. Moreover, a significant decrease of TEER to 48% of the initial value was measured within 24 h of LPS stimulation, which shows the negative effect on the barrier integrity. The calculated *P*_*app*_ values from the transport studies with the marker molecule sodium fluorescein indicated no difference between the stimulated and non-stimulated groups. The Caco-2 permeability assay is established as a “gold standard” when it comes to the prediction of human intestinal absorption because of its similarities (e.g., transporter, efflux pumps) to the intestinal epithelium [67] [68]. However, it is also reported that the Caco-2 cells form less permeable tight junctions than in vivo, which results in a low permeability for molecules that are transported mainly over the paracellular route [69] [70], which could be described as the main transport route for the small hydrophilic drug sodium fluorescein [71]. Additionally, the GI epithelium is classified as “leaky,” when TEER values of 50 to 100 Ω*cm^2^ are obtained [72], which indicates that the measured TEER values in this paper for the LPS control (200–350 Ω*cm^2^) were still too high to meet the KE of increased permeability, despite the significant decrease in comparison to the medium control (> 600 Ω*cm^2^). As a conclusion, transport studies with sodium fluorescein in the co-culture system showed that the *P*_*app*_ is not a predictive readout for the presented in vitro assay.

The second MIE is the first-line treatment drug for IBD recommended in “Guidelines for the management of inflammatory bowel disease in adults” mesalzine, a 5-aminosalicylic acid derivate (5-ASA), that is applied to cure mild inflammations (level 1) and to prolong the remission phase of the disease [13]. Mesalazine acts locally on the colonic mucosa and initiates various anti-inflammatory processes. It has been discovered that the main mode of action is the activation of the peroxisome proliferator activated receptor (PPAR) regulates the production of pro-inflammatory cytokines such as TNF-α, IL-6, IL-8, and IL-1, reduces the NF-κB activity, regulates the synthesis of prostaglandins and leukotrienes, and maintains the mucosal integrity [14, 73, 74]. The treatment of the LPS-induced inflammations of the in vitro assay resulted in a slight decrease of the TNF-α, IL-6, and IL8 release. The TEER values were increased to around 68% compared to the LPS control (48%) of the initial values. Therefore, the in vitro assay seems to be sensitive to the KE of mesalazine treatment concerning the readouts cytokine release and TEER.

The glucocorticoid (GC) prednisolone (severity level 2) binds to the glucocorticoid receptor (GR) (MIE 3) present in the cytoplasm. Based on the GC/GR interaction, which leads to conformational changes, the complex is translocated to the nucleus. The complex binds to DNA at specific glucocorticoid-responsive elements (GREs) and regulates the stimulation and suppression of numerous gene transcriptions such as the synthesis of pro-inflammatory cytokines and of the transcription factor NF-κB [15, 75, 76]. Moreover, prednisolone is known to decrease intestinal permeability [15, 77]. The treatment of the LPS-induced inflammation with prednisolone in the in vitro assay led to a decrease of TNF-α, IL-6, and IL-8 and increases the TEER values in comparison to the LPS control to around 82% of the initial values.

In moderate to severe disease progression in steroid-dependent IBD, the immunosuppressant 6-mercaptorpurine (6-MP) is administered to maintain the remission phase (level 3), as it is recommended in the European Crohn’s and Colitis Organisation (ECCO) Guidelines [10, 11]. In the intestinal mucosa and the liver, the prodrug 6-MP gets activated by the enzyme hypoxanthine phosphoribosyltransferase (HPRT) and is metabolized to 6-thioguanine nucleotides (6-TGNs) that act as purine analogues in DNA and RNA (MIE 4) [78]. This leads to an interference in numerous biological processes including the activation and apoptosis of immune cells, which results in immunosuppression [79–82]. The in vitro activation of 6-MP and azathioprine and the building of different thiopurine metabolites has recently been investigated by Genova et al., using the virus-immortalized human healthy colon cell line HCEC [83]. The treatment with 6-MP of the LPS-induced in vitro assay did not lead to a decrease of cytokine release for TNF-α, IL-6, and IL-8, which might indicate that the release of additional inflammatory mediators needs to be further investigated or longer incubation times and higher concentrations could be tested. Nevertheless, a strong barrier stabilizing effect of 6-MP (to 134% of initial value) on the LPS-inflamed MDM/Caco-2 co-culture was observed with TEER measurements.

The chimeric monoclonal TNF-α antibody infliximab is recommended by the ECCO in moderate to severe cases of IBD to induce clinical remission, when conventional therapy is unsuccessful (level 4) [10, 11]. Infliximab binds to the soluble and transmembrane form of TNF-α (MIE5), which results in the loss of bioactivity for soluble TNF-α in blood serum. It also induces apoptosis of immune cells such as T-cells and monocytes, resulting in a decrease of pro-inflammatory cytokine and chemokine production [84–86]. The findings of this investigation showed that after treatment of the LPS-induced inflamed co-culture with infliximab for 48 h, a significant decrease in the measured cytokine levels of TNF-α and IL-8, but not for IL-6 occured. The LPS-induced decrease in barrier integrity was treated successfully, which was indicated by TEER values of 92% after 48 h in comparison to the LPS control (48%). Accordingly, the effect of infliximab was detectable in the present in vitro assay with the readouts cytokine release (TNF-α, IL-8) and TEER, representing KE.

The calculation of drug efficacy for the readout parameter “cytokine release” showed that the present in vitro assay is able to simulate the effect for three out of four tested drugs on the release of the pro-inflammatory cytokines TNF-α, IL-6, and IL-8, whereas the effect of infliximab was limited to TNF-α and IL-8. As a result, there is a chance to identify active drug candidates with this readout (yes–no response), but the IVIVC might be poor as there are only macrophages present in the model. However, the predictivity of the in vitro assay might be improved by measuring further pro-inflammatory cytokines (e.g., IL-1 or IL-23). Regarding the readout TEER, the correlation of drug efficacy measured in the assay with the severity level of the disease (treatment pyramid) an R^2^ of 0.68 was recorded. This indicates a possible IVIVC for the readout TEER, which needs to be further investigated and verified by evaluating the efficacy of more drug candidates. As in vitro assay, a higher throughput is possible compared to animal experiments: one 12-well plate can be used for testing four drug candidates plus medium and LPS control (two wells, respectively), giving the possibility to repeat to the desired *n* of experiments. Performing one experiment took approximately 2 weeks (including cell seeding, MDM isolation stimulation, set-up of co-culture, inflammation treatment, TEER and cytokine release measurements, transport and ELISA studies). The acceptance criteria are a stable epithelial barrier of Caco-2 in the co-culture set-up (measured by TEER) and a significant decrease in LPS-induced epithelial integrity which is required to remain stable over the test period to ensure that a potential effect of drugs is measurable. For the proof of concept of the presented in vitro assay, FBS was used as a medium supplement to cultivate the cells. In the future characterization and validation of the assay, the FBS should be replaced by defined supplements to further reduce the pain and suffering of animals.

The in vitro assay represents different KE which can be compared to the KE observable in animal experiments. To compare the predictability of the in vitro assay to in vivo experiments, a comparison with the readouts obtained from IBD-related animal models was performed (see Supplementary information, Tables S1 and S2). One important readout for the commonly applied animal models such as the chemically IBD induced dextran sulfate sodium (DSS) model or the 2,4-dinitrobenzene-sulfonic acid (DNBS)/trinitrobenzene-sulfonic acid (TNBS) model is the disease activity index (DAI). Employing this score system, the symptoms of the diseased animals, for instance, rectal bleeding, weight loss, diarrhea, or anaemia, are rated according to their severity and are compared to the treated group [22, 87]. Epithelial erosions and ulcerations, crypt abscess, infiltration of immune cells, and tumour formation (for TNBS/DBNS models) are rated with the histology injury score (HIS) [21, 22]. These organism responses cannot be simulated with in vitro models. In contrast, the activation of inflammatory pathways, as investigated in animal models, is able to be analysed with the in vitro model by measuring pro-inflammatory cytokine release. Additionally, the assessment of the barrier function in vivo gives the potential to be addressed by TEER measurements in vitro. Consequently, the in vitro assay represents two important KE: cytokine profile and barrier integrity, which might link the in vivo situation represented by animal models.

In recent years, in vitro co-culture models have regularly been applied to test the anti-inflammatory properties of compounds, for instance, budesonide nanoformulations, cinnamon extracts, or natural marine organism products with readouts such as TEER, cytokine release, m-RNA level, and NF-κB activity [54, 88, 89]. Dooley et al*.* further studied the correlation of gene expression between IBD tissue samples and Caco-2 cells, which were treated with IBD drugs to identify potential biomarkers [90]. However, it is very important to evaluate the IVIVC of the individual readouts to optimize the predictability of the in vitro models in order to finally have the opportunity of validation and standardization regarding “Good Cell and Tissue Culture Practice (GCCP)” and to ideally achieve regulatory application.

There are numerous requirements that need to be considered when standardizing in vitro cell models to achieve a high reproducibility and predictivity. First, crucial factors that affect the cell culture system need to be identified. Therefore, the origin of the cell line and its phenotype/genotype, the purity, and culture stability should be confirmed. Furthermore, the influence of reagents such as medium additives and antibiotics and the environment of the cells needs to be investigated [91]. In literature, it is reported that the composition of medium, passage number, culture time, and the culturing system itself are important factors for the cultivation of Caco-2 cells, which have an impact on the differentiation and permeability of the cell layer [92]. Moreover, for the Caco-2 cell line, different sub-populations are reported, which differ in morphology [93]. Natoli et al*.* additionally identified that the confluency has also an influence on the polarization and homogeneity of the cells [94]. MDM are known to have a heterogeneous phenotype. Microbial or environmental factors can lead to variations in their phenotype [95]. In addition, the stimuli utilized for differentiation such as bacterial agents, cytokines or chemokines [96], or colony stimulating factors [97] have an impact on the differentiation either in the M1 or M2 type, which emphasizes the need for identity control. Based on these observations, the definition of culture condition and of the culture protocols for both the Caco-2 cells and MDM are essential for standardizing the in vitro co-culture. Besides that, the MDM show a donor dependency, which is particularly noticeable in the wide variations in TNF-α release [98] and were also present in our experiments. This characteristic requires the setting of acceptance criteria in the standardization process that need to be fulfilled by the MDM used for the co-culture experiments [5].

Secondly, the implementation of a quality management (QM), consisting of quality assurance (QA) and quality control (QC), is required to ensure consistent integrity, validity, and reproducibility [91]. The way of implementation and the exact standard operation procedures (SOPs) depend on the laboratory and the research project. However, the “Guidance Document on Good In Vitro Method Practices (GIVIMP)” already describes required key aspects such as quality control of materials used, the laboratory environment and equipment, defining acceptance criteria as well as ensuring the competence of the experimenter [99]. In the next step, the documentation system should be focused to track the materials and methods that are used for performing the in vitro assay. Moreover, risk assessment is necessary to develop measures to protect the environment and individuals from potential hazards. Furthermore, the compliance with regulations and laws has to be investigated and adequate education and training for the persons, that perform the cell experiments, must be provided [91]. In conclusion, the standardization process is a future challenge that should be addressed to ensure high reproducibility and predictivity of the in vitro assay.

## Conclusion

The development of an in vitro assay for the screening of novel IBD-related drugs with a relevant human output formed the core focus. The readouts cytokine release, TEER, and *P*_*app*_ of sodium fluorescein were investigated in terms of their predictability; pharmaceuticals with known human efficacy in IBD were used as controls. The assay was evaluated in the context of the “Guidance Document For Describing Non-Guideline In Vitro Test Methods.” Leading to the creation of an AOP/EOP framework to describe the KE relevance for the in vitro assay. The readout P_app_ calculated from transport studies with sodium fluorescein could not simulate the KE; in contrast, the assay successfully represented the KE of LPS stimulation regarding cytokine release and TEER. Likewise, the readout TEER showed a comparably high IVIVC of *R*^2^ = 0.68 calculated by drug efficacy related to the severity level represented by the evaluated drugs. The present in vitro assay provides a good platform for additional investigations. Further drug candidates need to be tested to improve the reliability of the in vitro assay and also standardizing of the in vitro assay is fundamentally to open up the opportunity to reduce animal experiments in drug testing.

## Supplementary Information

Below is the link to the electronic supplementary material.

Supplementary file1 (DOCX 1.69 MB)

## Data Availability

The datasets generated during and/or analysed during the current study are available from the corresponding author on reasonable request.
