# Supplementary material for: Inflammatory bowel disease addressed by Caco-2 and monocyte-derived macrophages: an opportunity for an in vitro drug screening assay
Source: In Vitro Model. 2022 Nov 3;1(4-5):365–83. doi: 10.1007/s44164-022-00035-8 (PMC9630817; doi:10.1007/s44164-022-00035-8)
Supplement: Supplementary file 1 — Supplementary file1 (DOCX 1.69 MB) [file 44164_2022_35_MOESM1_ESM.docx]

**Supplementary information**

**Investigation of the epithelial barrier**

The epithelial barrier of the Caco-2 monolayer with TEER > 500 Ω*cm^2^ was visualized by staining of the barrier forming protein zonula occludens and the cell nuclei and imaging via confocal laser scanning microscopy (Figure S1). The reaction of the epithelial cells to an inflammatory stimulus was tested by adding TNF-α in different concentrations and the effect on the TEER values were measured (Figure S2).

**
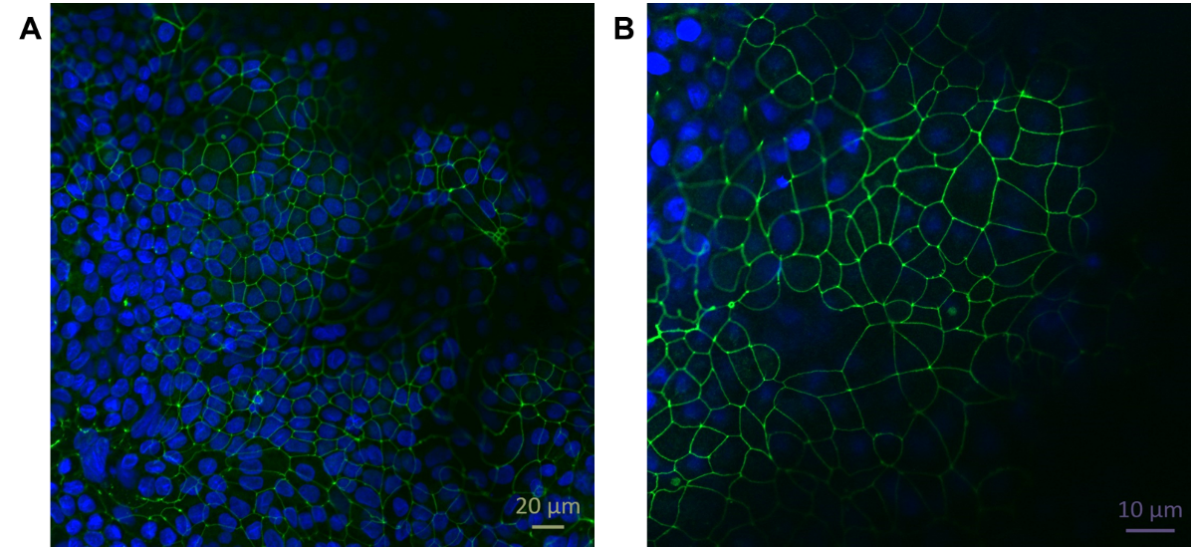
**

**Figure Supplementary 1** Staining of the tight junction protein Zonula occludens-1 and the nuclei with ZO-1 antibody and DAPI of the Caco-2 monolayer, showing the developed epithelial barrier. The cells were imaged via confocal laser scanning microscopy


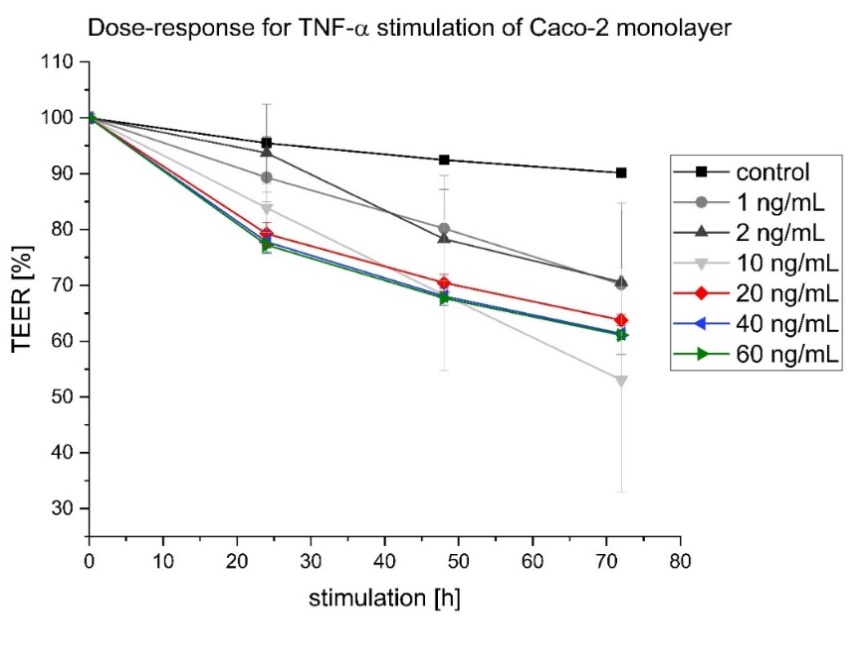


**Figure Supplementary 2** Dose-dependent decrease of TEER after stimulation of Caco-2 cells with different TNF-concentrations from 1 ng/mL to 60 ng/mL. The highest effect could be observed by stimulation with 10 ng/mL. Results are represented as mean ± SD for in summary n=9 wells for each group performed in three biological replicates

**Cytokine release of MDM after incubation with LPS**

The release of TNF-α, IL-6 and IL-8 by the MDM after LPS-stimulation was analysed by performing ELISA measurements (Figure S3)


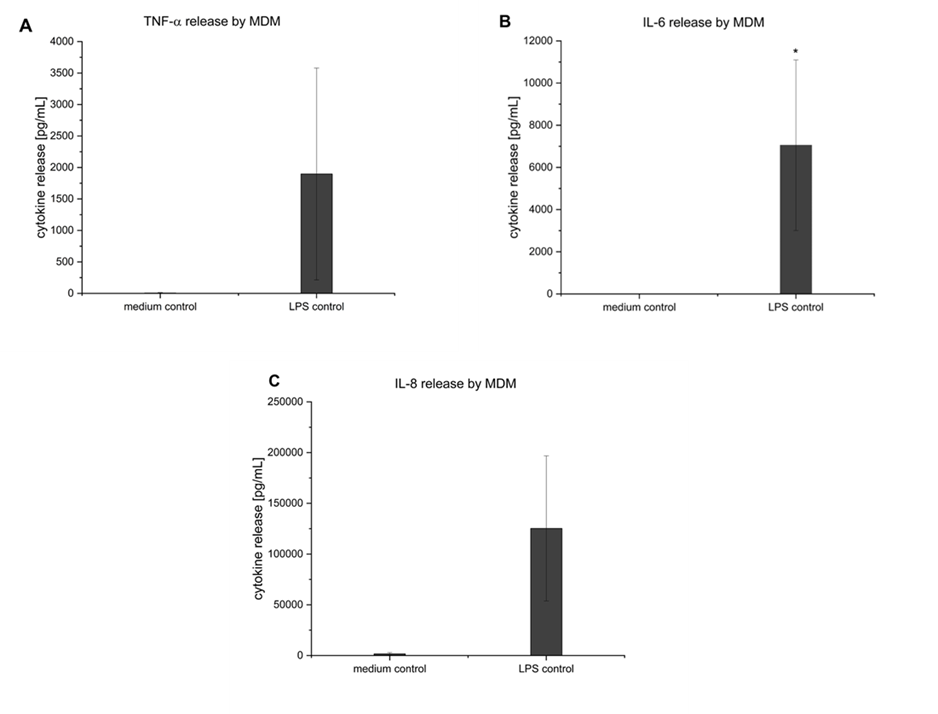


**Figure Supplementary 3** LPS-induced cytokine release of TNF-IL-6 and IL-8 by MDM after 24 h of incubation. An increase in the cytokine release could be observed, but also a donor-dependency. Results are represented as mean ± SD for in summary n=6 wells for each group performed with supernatants out of three biological experiments. *p < 0.05, **p< 0.01 and ***p< 0.001 indicate significant difference

**Cell viability after incubation with APIs in different concentrations**

The cytotoxicity of the applied drug concentration in the co-culture experiments (= 200 µg/mL) was excluded by performing MTT assays for the Caco-2 cells and the MDM (figure S4 and S5).


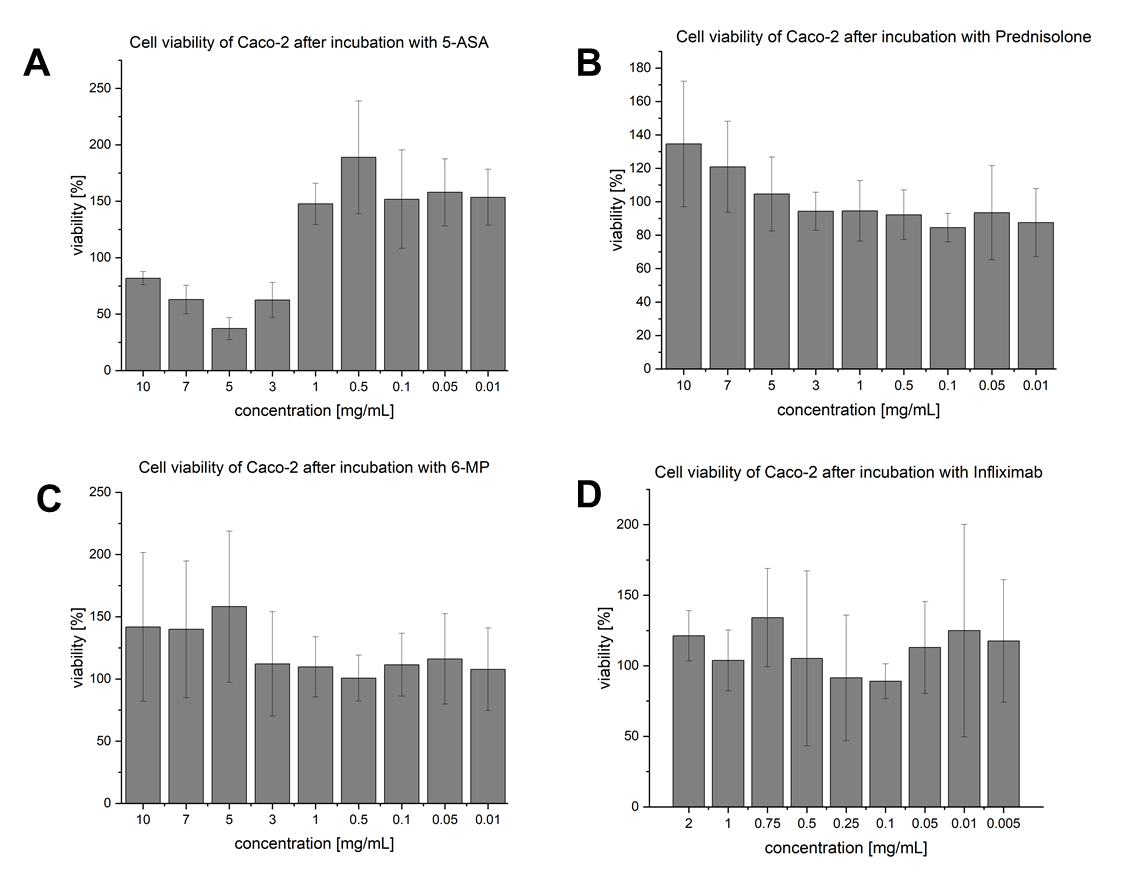


**Figure Supplementary 4** Cell viability of Caco-2 cells for different concentrations of the tested drugs Mesalazine, Prednisolone, 6-Mercaptopurine and Infliximab investigated by MTT assays. Results are represented as mean ± SD for in summary n=9 wells for each concentration performed in three biological replicates


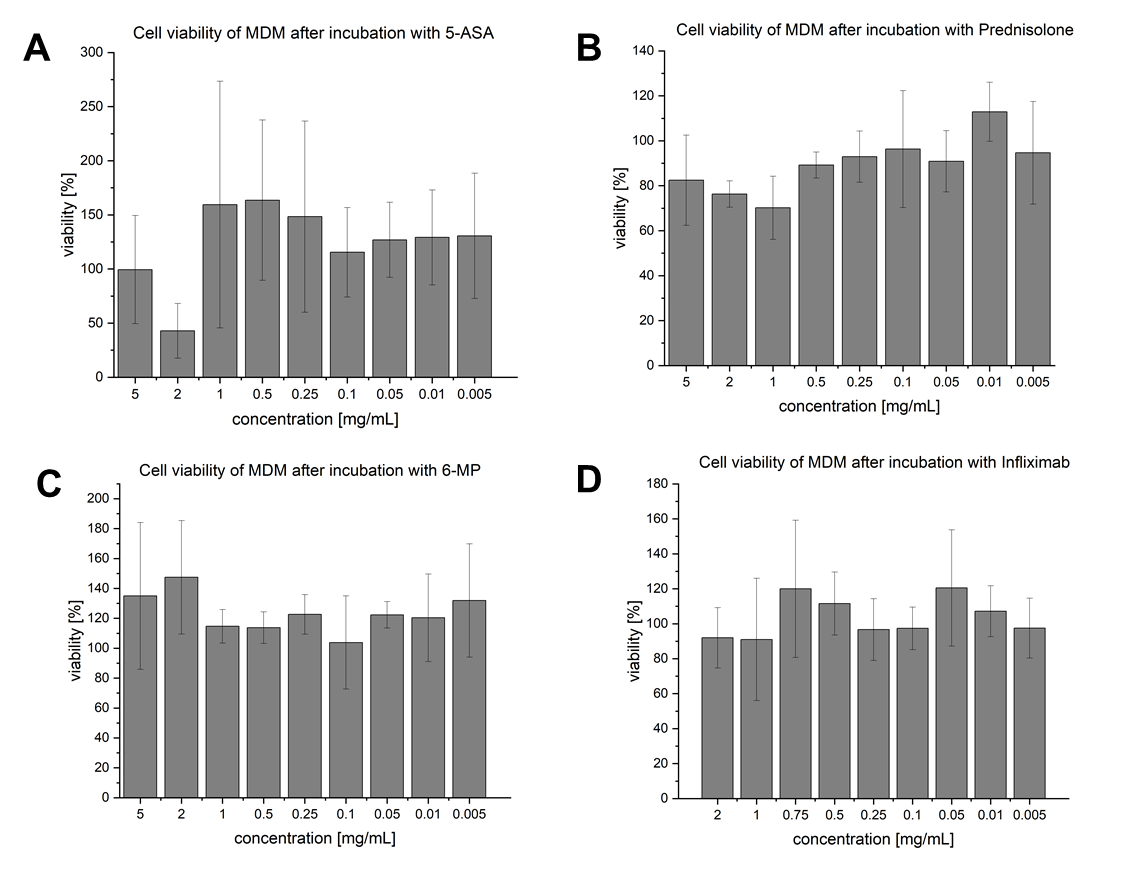


**Figure Supplementary 5** Cell viability of MDM for different concentrations of the tested drugs Mesalazine, Prednisolone, 6-Mercaptopurine and Infliximab investigated by MTT assays. Results are represented as mean ± SD for in summary n=9 wells for each concentration performed in three biological replicates

**Investigation of barrier permeability for sodium fluorescein**

The permeability of the Caco-2 cells in the co-culture system was analysed after the stimulation with LPS and the treatment with the four different drugs. Transport studies with sodium fluorescein were performed and the *P_app_* values were calculated (Figure S6).


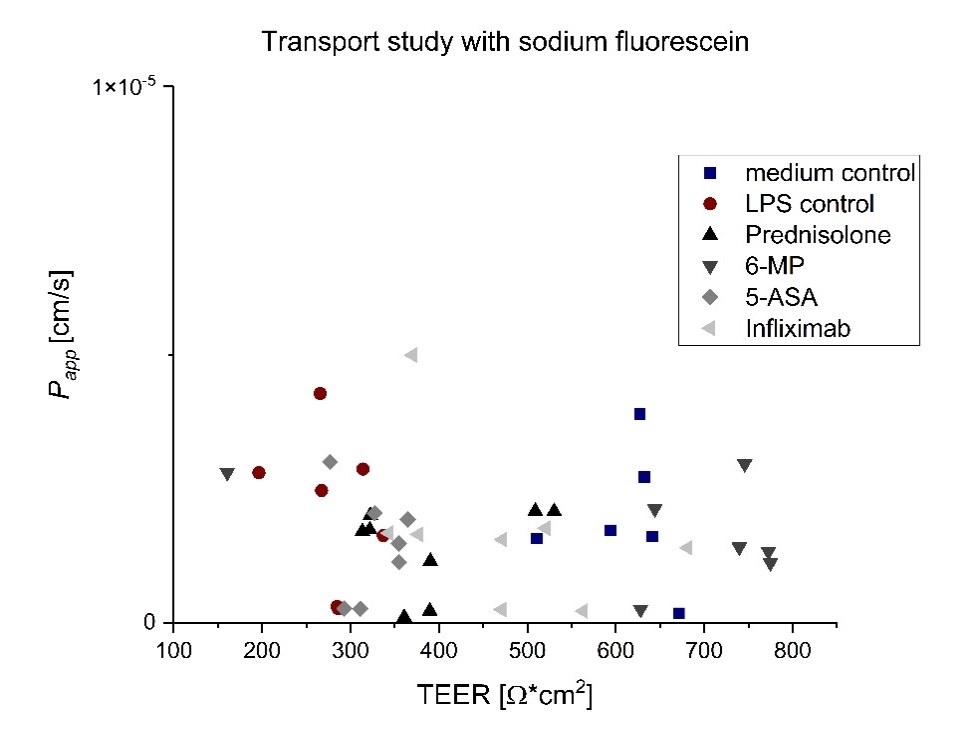


**Figure Supplementary 6 Effect on barrier integrity investigated by calculated *P_app_* values after the performance of transport studies**. The experiment was done with sodium fluorescein after treatment with the drugs 5-ASA, Prednisolone, 6-MP and Infliximab with sodium fluorescein**.** No meaningful differences for the *P_app_* values of the respective groups could be observed indicating no increased permeability of the epithelial barrier. In summary n=6 wells for medium control and n=8 wells for the other groups, performed in four biological replicates

**Comparison of the *in vitro* readouts to the readouts of IBD-related animal models**

IBD-related animal models are used for the investigation of safety and efficacy of new potential drugs in preclinical studies. To evaluate the predictability of the *in vitro* assay to the *in vivo* situation, a comparison with the readouts of IBD-related animal models was performed and is shown in the following table 1.

**Table Supplementary 1** Overview of commonly used IBD-related animal models and their readouts, which can be partially investigated with the here presented *in vitro* assay

| **Model** | **Inducer** | **Reference** | **Readout** | **achievable with *in vitro* assay** |
| --- | --- | --- | --- | --- |
| IBD mouse/rat or guinea pig model | **chemically induced**:  Dextran Sulfate Sodium (DSS) | [21],[22] | **clinical disease activity score**: rectal bleeding, weight loss, diarrhoea, anaemia  **histology injury score (HIS)**: epithelial erosion and ulceration, crypt abscess, infiltration of immune cells, tumour formation (for TNBS/DNBS models)  **biomarkers/cytokine release**: activation of inflammatory pathways | no  no  yes |
| IBD rat or mouse model | **chemically induced**:  2,4-Dinitrobenzene-sulfonic acid (DNBS)/ Trinitrobenzene-sulfonic acid (TNBS)-Induced Colitis | [20],[87] |  |  |
| Cytokine knock-out animals | **genetically engineered**: *e.g.*, IL-2 or IL-10 knock-out mice | [25],[23], [100] | **intestinal permeability**: assessment of barrier function  **systemic endotoxemia**: labelling with specific antibodies  **histology injury score** (HIS): inflamed mucosa with crypt abscess, degenerative lesions of epithelium, infiltration of immune cells  **biomarkers/cytokine release**: activation of inflammatory pathways | yes  no  no  yes |
| Transgenic models  **Genetically engineered** | **genetically engineered**:  genetic modification in the genome  *e.g.*, IL-7 transgenic mice with overexpression of IL-7 mRNA or (STAT)-4 transgenic mice with a knock-out of STAT-4 | [101],[102] | **clinical disease activity score**: rectal bleeding, weight loss, diarrhea, rectal prolapse (only IL-7 transgenic mice)  **histology injury score** (HIS): inflamed mucosa with crypt abscess, erosions, wall thickening, goblet cell-depleted epithelium, infiltration of immune cells  **biomarkers/cytokine release**: activation of inflammatory pathways | no  no  yes |
| Severe combined immuno-deficiency (SCID)-mice | **immune-mediated/adoptive transfer**:  *e.g.*, transfer of CD4^+^CD45RB^High^ cells or CD62L+ CD4+ T cells | [103], [104] |  |  |
| Rag-knock-out mice | **immune-mediated/adoptive transfer**: transfer of syngeneic T lymphocytes (*e.g.,* Con A CD4+ T cell blasts) to Rag-1 or Rag-2 knock-out mice | [105] |  |  |
| SAMP1/Yit model | **spontaneous (inbred) mediated:**  spontaneous development of terminal ileitis with typical features of CD at age of 10 weeks | [106] |  |  |
| C3H/HeJBir model | **spontaneous (inbred) mediated:**  spontaneous development of colitis in the cecum and right colon at age of 3-4 weeks (depending on environmental factors) | [107] |  |  |

**Table Supplementary 2** Overview of drug efficacy observed in animal models and the efficacy that was measured in the *in vitro* assay

| **API** | **Animal model** | ***In vitro* model** |
| --- | --- | --- |
| Mesalazine (5-ASA)  (level 1) | **rat DSS/TNBS-model (intracolonic administration of 100 mg/kg per day)**  [108,109]  **measurable effects:**   - preventing of colon shortening - inhibition of white blood cell increase (to 61% compared to inflamed group [108]) - decrease of histology injury score (46% [108] and 32% [109]) - decrease of tissue myeloperoxidase activity (to approximately 67% [108] and 56% [109] compared to inflamed group) - decrease of mucosal permeation - restoring of the typical reticular pattern of TJ protein occludin - no significant decrease in cytokine release (IL-1, IL-4, IL-6 and TNF-α) | **basolateral treatment**  **(200 µg/mL)**  **measurable effects:**   - stabilizing of LPS-induced decrease in TEER to 67% of the initial value - significant decrease in IL-8 release - no significant decrease in cytokine release for IL-6 - decrease in TNF-α release, but not significant (due to the high standard deviation of the LPS group) |
| Prednisolone  (level 2) | **rat TNBS model (intracolonic administration of 2 mg/kg per day)**  [110]  **measurable effects:**   - reduction of diarrhoea, adhesions, apoptosis - decrease of macroscopic damage score to approx. 75 % - decrease of histology injury score (to approx. 56 % compared to inflamed group) - recovery of intestinal cytoarchitecture - decrease of tissue myeloperoxidase activity (to approx. 64% compared to inflamed control) - reduction in infiltration of inflammatory cells - significant decrease in TNF-α release, in contrast to IL-1 beta release | **basolateral treatment**  **(200 µg/mL)**  **measurable effects:**   - stabilizing of LPS-induced decrease in TEER to 82% of the initial value - significant decrease in IL-8 and IL-6 release - decrease in TNF-α release, but not significant (due to the high standard deviation of the LPS group) |
| 6-Mercaptopurine  (level 3) | **mouse DSS-model (orally administration of 0.5, 1 and 2 mg/kg 6-TG per day)**  [111]  **measurable effects:**   - preventing of colon shortening - decrease of Disease Activity Index - decrease of histology injury score to 58% - decrease of tissue myeloperoxidase activity (to approx. 65% compared to inflamed control) | **basolateral treatment**  **(200 µg/mL)**  **measurable effects:**   - stabilizing of LPS-induced decrease in TEER to 134% of the initial value - no significant decrease in cytokine release for TNF-α, IL-6 and IL-8 |
| Infliximab  (level 4) | **rat TNBS model (intracolonic administration of 1, 3 or 5 mg/kg per day)**  [112,113]  **measurable effects:**   - decrease of macroscopic damage score - decrease of microscopic damage score - reduction in infiltration of inflammatory cells - decrease of tissue myeloperoxidase activity (to approx. 39% compared to inflamed control) - increase of IL-10 gene expression in mesenteric adipose tissue - significant decrease of TNF-α level in serum and tissue [113] | **basolateral treatment**  **(200 µg/mL)**  **measurable effects:**   - stabilizing of LPS-induced decrease in TEER to 92% of the initial value - significant decrease in IL-8 release, no effect on IL-6 release - increase in IL-10 release, but not significant due to the high standard deviation of the LPS group and low amounts in general) - no release of TNF-α measurable after treatment |

**References**

101. Watanabe M, Ueno Y, Yajima T, Okamoto S, Hayashi T, Yamazaki M, Iwao Y, Ishii H, Habu S, Uehira M, Nishimoto H, Ishikawa H, Hata JI, Hibi T. Interleukin 7 transgenic mice develop chronic colitis with decreased interleukin 7 protein accumulation in the colonic mucosa. J Exp Med. 1998;187:389–402. https:// doi. org/ 10. 1084/ jem. 187.3. 389.

102. Wirtz S, Neurath MF. Animal models of intestinal inflammation: new insights into the molecular pathogenesis and immunotherapy of inflammatory bowel disease. Int J Colorectal Dis. 2000;15:144–60. https://doi.org/10.1007/s003840000227.

103. Powrie F, Leach MW, Mauze S, Caddie LB, Coffman RL. Phenotypically distinct subsets of cd4+t cells induce or protect from chronic intestinal inflammation in c. B-17 scid mice. Int Immunol. 1993;5:1461–71. https://doi.org/10.1093/ intimm/5.11.1461.

104. Mudter J, Wirtz S, Galle PR, Neurath MF. A new model of chronic colitis in SCID mice induced by adoptive transfer of CD62L+ CD4+ T cells: insights into the regulatory role of interleukin-6 on apoptosis. Pathobiology. 2003;70:170–6. https://doi.org/10.1159/000068150.

105. Trobonjaca Z, Leithäuser F, Möller P, Bluethmann H, Koezuka Y, MacDonald HR, Reimann J. MHC-II-independent CD4 + T cells induce colitis in immunodeficient RAG −/− hosts. J Immunol. 2001;166:3804–12. https://doi.org/10.4049/jimmunol.166.6.3804.

106. Matsumoto S, Okabe Y, Setoyama H, Takayama K, Ohtsuka J, Funahashi H, Imaoka A, Okada Y, Umesaki Y. Inflammatory bowel disease-like enteritis and caecitis in a senescence accelerated mouse P1/Yit strain. Gut. 1998;43:71–8. https://doi.org/10.1136/gut.43.1.71.

107. Elson CO, Cong Y, Sundberg J. The C3H/HeJBir mouse model: a high susceptibility phenotype for colitis. Int Rev Immunol.2000;19:63–75. https://doi.org/10.3109/08830180009048390.

108. Hayashi Y, Aoyagi K, Morita I, Yamamoto C, Sakisaka S. Oral administration of mesalazine protects against mucosal injury and permeation in dextran sulfate sodium-induced colitis in rats. Scand J Gastroenterol. 2009;44:1323–31. https://doi.org/10.3109/00365520903262414.

109. Siddiqui A, Ancha H, Tedesco D, Lightfoot S, Stewart CA, Harty RF. Antioxidant therapy with N-acetylcysteine plus mesalamine accelerates mucosal healing in a rodent model of colitis. Dig Dis Sci. 2006;51:698–705. https://doi.org/10.1007/s10620-006-3194-z.

110. Witaicenis A, Luchini AC, Hiruma-Lima CA, Felisbino SL, Garrido-Mesa N, Utrilla P, Gálvez J, Di Stasi LC. Suppression of TNBS-induced colitis in rats by 4-methylesculetin, a natural coumarin: comparison with prednisolone and sulphasalazine. Chem Biol Interact. 2012;195:76–85. https://doi.org/10.1016/j.cbi.2011.11.004.

111. Sann H, Von Erichsen J, Hessmann M, Pahl A, Hoffmeyer A. Efficacy of drugs used in the treatment of IBD and combinations thereof in acute DSS-induced colitis in mice. Life Sci. 2013;92:708–18. https://doi.org/10.1016/j.lfs.2013.01.028.

112. Clemente TRL, Dos Santos AN, Sturaro JN, Gotardo ÉMF, De Oliveira CC, Acedo SC, Caria CREP, Pedrazzoli J, Ribeiro ML, Gambero A. Infliximab modifies mesenteric adipose tissue alterations and intestinal inflammation in rats with TNBS-induced colitis. Scand J Gastroenterol. 2012;47:943–50. https://doi.org/10.3109/00365521.2012.688213.

113. Woodruff TM, Arumugam TV, Shiels IA, Reid RC, Fairlie DP, Taylor SM. A potent human C5a receptor antagonist protects against disease pathology in a rat model of inflammatory bowel disease. J Immunol. 2003;171:5514–20. https://doi.org/10.4049/jimmunol.171.10.5514.
